# The *fruB* Gene of Streptococcus mutans Encodes an Endo-Levanase That Enhances Growth on Levan and Influences Global Gene Expression

**DOI:** 10.1128/spectrum.00522-22

**Published:** 2022-05-19

**Authors:** Brinta Chakraborty, Lin Zeng, Robert A. Burne

**Affiliations:** a Department of Oral Biology, University of Florida College of Dentistry, Gainesville, Florida, USA; Emory University School of Medicine

**Keywords:** exopolysaccharide, transcriptional regulation, enzymatic activity, dental caries, biofilms

## Abstract

Streptococcus mutans, the primary etiologic agent of human dental caries, and a variety of oral Streptococcus and Actinomyces spp. synthesize high molecular mass homopolymers of fructose (fructans) with predominantly β2,1- (inulins) or β2,6-linkages (levans). The ability of S. mutans to degrade fructans contributes to the severity of dental caries. The extracellular product of *fruA* of S. mutans is an exo- β-d-fructofuranosidase that releases fructose from levan and inulin. Located 70 bp downstream of *fruA, fruB* encodes a member of the glycoside hydrolase family 32, but the function of FruB has not been established. Growth assays performed using wild-type UA159 and *fruB-*deficient derivatives, with fructans as the sole carbohydrate source, showed a significant reduction in the growth rate of a *fruB* mutant on levan, but not on inulin. A purified, recombinant FruB protein degraded levan to release mainly fructooligosaccharides. Driven by the *fruA* promoter and a secondary promoter located in the 3′ region of the *fruA* sequence, the *fruB* gene is inducible by fructose and especially by levan, but a stable stem-loop structure in the intergenic region likely modulates transcriptional read-through from *fruA*. Transcriptomic analysis of UA159 and a *fruB* mutant grown on 0.2% levan revealed differential expression of genes encoding ABC transporters, transcriptional regulators and genes involved in growth and stress tolerance. The ability of FruB to enhance levan metabolism and the high degree of conservation of FruB across S. mutans isolates imply a significant contribution of FruB to the fitness and virulence of this pathogen in human dental biofilms.

**IMPORTANCE** Carbohydrate metabolism and acid production are essential for the development of dental caries. As a by-product of sucrose metabolism, formation, and degradation of fructans enhances the severity of caries by S. mutans in animal models. This study highlights a significant breakthrough in identifying FruB in S. mutans as an endolevanase that contributes to efficient utilization of levan, a specific type of fructan produced by certain commensals but not S. mutans. Transcriptomic analysis revealed that FruB-dependent levan metabolism impacted global gene regulation, including a large number of novel genes. Considering the preference for levan by both FruA and FruB, the conservation of *fruAB* in S. mutans might represent a competitive advantage in access to the energy storage produced by dental microbiome. This is the first report demonstrating the presence of an endolevanase in S. mutans, therefore should be of broad interest to the fields of dental caries and complex carbohydrate metabolism.

## INTRODUCTION

The resident microbiota of the human oral cavity experiences frequent and substantial environmental and nutritional fluctuations associated with the intermittent eating patterns and diurnal rhythms of the host. The rapid changes in the availability and source of carbohydrates has a profound impact on the oral microbiome and, thus, many members of the oral microbiota have evolved numerous strategies to optimize utilization of carbohydrates (1). Lactic acid bacteria (LAB), such as S. mutans, rely on carbohydrate metabolism as the primary source for energy production (2). S. mutans is a primary etiological agent of dental caries in humans, attributable in large part to its efficiency in forming biofilms, its capacity to ferment many different carbohydrates and its ability to synthesize and hydrolyze extracellular polymers of d-glucose and d-fructose, commonly known as glucans and fructans, respectively (3, 4). While glucans act as an adhesive scaffolding to promote the formation of oral biofilms, the ability of this cariogenic bacterium to produce and degrade fructans provides a carbon and energy reserve during periods of nutrient starvation, prolongs production of organic acids through glycolysis, and contributes directly to caries formation (5).

Many oral streptococci and some *Actinomyces* spp. produce fructans using fructosyltransferase (FTF) enzymes, with dietary sucrose as the substrate (6). Structural analysis of fructans revealed that *S. salivarius* and *Actinomyces* spp. produce levan-type fructans (mostly β2,6-linked with some β2,1 branching), whereas S. mutans produces inulin-type fructans rich in β2,1 linkages (7). Organisms that synthesize fructans generally express at least one fructan hydrolase enzyme (fructanase), including the oral bacteria that produce the majority of fructans in human oral biofilms. Further, depending on the organism and particular gene product, the fructanase enzymes can act endo- or exo-hydrolytically, displaying various levels of specificity for levans or inulins, and some can cleave the fructose moiety from the trisaccharide raffinose and the disaccharide sucrose (8). Fructans accumulate rapidly in the dental biofilms of humans after ingestion of sucrose, consistent with their high molecular mass (10^6^ to 10^7^ Daltons) and resultant slow diffusion characteristics (6, 9). These fructans then disappear over the course of the ensuing 30 to 60 min due, in large part, to the action of fructanases (10, 11). Consistent with the observations of fructan accumulation and disappearance in human dental plaque and coupled with experiments conducted with strains of S. mutans with defects in fructan metabolism (5), there is a general agreement that fructans serve mainly as extracellular storage polysaccharides that can be hydrolyzed by fructanases when readily available carbohydrate sources are cleared from the oral cavity.

The primary fructanase enzyme of S. mutans, encoded by the *fruA* gene, has been purified and characterized, and the gene itself has served as an excellent model to reveal novel regulatory circuits that control the induction of gene expression by carbohydrates and peculiarities of carbohydrate catabolite repression (CCR) in S. mutans (12, 13). FruA is a 140 kDa, secreted exo-β-D fructosidase with a strong preference for levans, but the enzyme is also highly active against inulins, fructooligosaccharides (FOS), sucrose and raffinose. Thus, far, FruA appeared to be the sole enzyme involved in utilization of extracellular fructose polymers by S. mutans (7, 14), as inactivation of *fruA* in strains UA159 and GS-5 eliminated the ability of the organism to grow on fructans. Previously, our group reported that expression of *fruA* was inducible by growth on levans and inulins and was exquisitely sensitive to carbon catabolite repression (CCR) (4). In S. mutans, expression of *fruA* is repressed in the presence of mM concentrations of preferred hexoses (e.g., glucose) and is optimal when the cells are grown in levans or inulins, which provide steady-state levels of fructose that serve as the inducing signal (4, 13, 15). The presence of a dyadic sequence positioned at -72 to -59 relative to the transcription start site (TSS) is required for *fruA* expression and is likely the target for the LevR transcriptional activator, which is a response regulator that is part of an unusual four-component signal transduction pathway (LevQRST) required for activating *fruA* in response to fructose (13). Homologues of FruA were identified in related oral bacteria, such as *S. salivarius* (16), and S. gordonii (17); each as the sole likely contributor of their fructanase activities.

In strains of S. mutans, the *fruA* gene is almost always located in an apparent operon with a downstream gene, *fruB*, that is predicted to encode a 57 kDa secreted protein with significant similarity to bacterial β-fructosidases (15% identical and 29% similar to FruA), although no biochemical activity has yet been ascribed to this putative protein (13). FruB is predicted to be a member of the glycoside hydrolase family-32 enzymes, which catalyze the hydrolysis of various glycosidic bonds in complex polysaccharides (18). The primary sequence of FruB is also highly conserved in most S. mutans isolates, but no direct role of FruB in fructan metabolism has been established. The objectives of the current study were to investigate the function of FruB in the context of fructan metabolism and its overall impact on bacterial gene expression, as well as to obtain preliminary insights into how *fruB* may be regulated. The study established how FruB may be integrated into the utilization of storage exopolysaccharides by a prominent caries pathogen.

## RESULTS

### Strains lacking fructan-hydrolyzing enzymes exhibit slow growth and lower final yield on levan.

**FIG 1:**
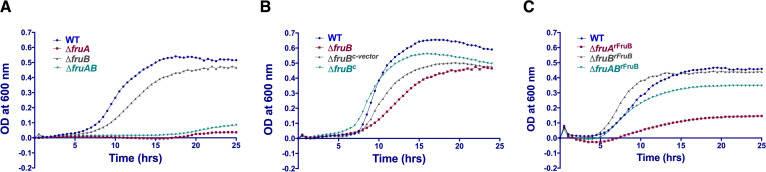
Growth curves of S. mutans wild type and mutant strains with levan as the sole carbohydrate source. (A) Growth of the WT UA159 and mutants deficient in *fruA*, *fruB* or both. (B) A WT copy of *fruB* gene was cloned into a shuttle vector pIB184 and the resultant plasmid was introduced into △*fruB* (△*fruB^c^*). An empty pIB184 was introduced into △*fruB* background to serve as a vector control (Δ*fruB^c-vector^*). (C) WT and three mutant strains were supplemented with purified 100 nM recombinant protein rFruB. Data are representative of three independent experiments performed at least in triplicate.

Previous efforts in our laboratory to establish a function for the *fruB* gene product, mainly focused on the use of commercially available fructans, did not yield definitive results. To investigate the role of one or both frucatanase genes during the growth of S. mutans on levan as the carbohydrate source, mutant strains were created employing marker-dependent polar and non-polar approaches (Table 1). In tryptone-vitamin (TV) medium supplemented with 0.2% (wt/vol) levan, synthesized using an FTF enzyme preparation from S. salivarius, wild-type S. mutans strains grew with a doubling time (Td) = 100 ± 3.4 min, whereas the Δ*fruB* strain consistently exhibited a longer lag phase, along with significantly slower exponential growth (Td = 125 ± 6.2 min) (*P < *0.0005) and lower final yields (*P < *0.05) (Fig. 1A). The Δ*fruA* mutant and Δ*fruAB* double mutant did not grow, even after 24 h. When grown in BHI, with glucose as the primary carbohydrate, or in TV medium formulated with glucose or fructose as the carbohydrate source, no differences in growth of any strains were noted (Fig. S1 in the supplemental material). Importantly, when cells were grown in the presence of inulin (from dahlia tubers) (primarily β2,1-linked fructans), no differences were observed between the wild type and the strain lacking only *fruB* (Fig. S1), indicative of a specific role for FruB in levan metabolism.

**TABLE 1:** Strains and plasmid used in this study^*a*^

Strains or plasmid	Relevant characteristics	Source or reference
S. mutans		
UA159	Wild-type	Laboratory stock (ATCC700610)
* ΔfruA*	*fruA:: Kan*	This study
*ΔfruB*	*fruB:: Kan*	This study
*ΔfruAB*	*fruAB:: Kan,* by introducing a polar kanamycin (Ω kan) mutation in *fruAB* operon by allelic exchange.	This study
*ΔfruA::pIB184*	*ΔfruA* harboring pIB184, Em^r^	This study
*ΔfruB::pIB184*	*ΔfruB* harboring pIB184, Em^r^	This study
*ΔfruAB:pIB184*	*ΔfruAB* harboring pIB184, Em^r^	This study
*ΔfruA::pIB184::fruB*	*ΔfruA* harboring pIB184::*fruB*, Em^r^	This study
*ΔfruB::pIB184:fruB*	*ΔfruB* harboring pIB184:: *fruB*, Em^r^	This study
*ΔfruAB::pIB184:fruB*	*ΔfruAB* harboring pIB184:: *fruB*, Em^r^	This study
CAT01	*veg* promoter and RBS of *fruB* transcriptionally fused with *cat* and integrated within UA159 chromosome via the vector pJL84, Kan^r^	This study
CAT02	*veg* promoter and *igr* of *fruAB* operon transcriptionally fused with *cat* and integrated within UA159 chromosome via the vector pJL84, Kan^r^	This study
CAT03	*fruA* promoter and RBS of *fruB* transcriptionally fused with *cat* and integrated within UA159 chromosome via the vector pJL84, Kan^r^	This study
CAT04	*fruA* promoter and *igr* of *fruAB* operon transcriptionally fused with *cat* and integrated within UA159 chromosome via the vector pJL84, Kan^r^	This study
CAT05	3′ end of *fruA* and RBS of *fruB* transcriptionally fused with *cat* and integrated within UA159 chromosome via the vector pJL84, Kan^r^	This study
CAT06	3′ of *fruA* and *igr* of *fruAB* operon transcriptionally fused with *cat* and integrated within UA159 chromosome via the vector pJL84, Kan^r^	This study
CAT07	*igr* of *fruAB* operon transcriptionally fused with *cat* and integrated within UA159 chromosome via the vector pJL84, Kan^r^	This study
Plasmids		
pIB184	Shuttle expression plasmid with constitutive P23 promoter, Em^r^	(34)

a Kan^r^, resistant to Kanamycin; Em^r^, resistant to erythromycin. Constructs containing the *cat* gene were synthesized using GBlocks (Integrated DNA Technologies, IA, USA).

When strain Δ*fruB* was complemented with plasmid pIB184 (19) carrying an intact *fruB* driven by the P_23_ promoter, the growth defect on levan was partially restored, supporting that the defect in growth on levan by the *fruB* mutant strain was attributable to loss of FruB (Fig. 1B).

### FruB is a secreted protein.

**FIG 2:**
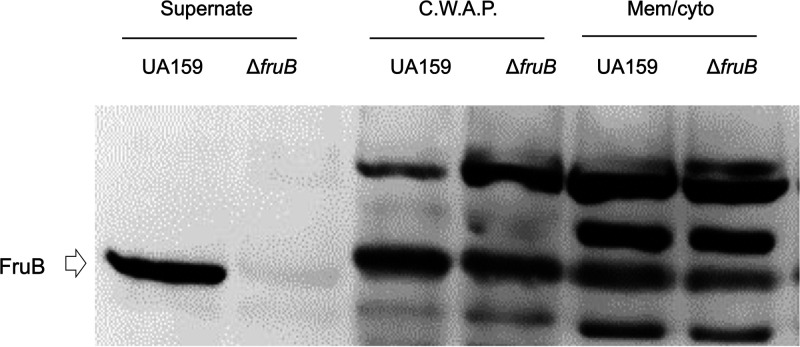
Localization of FruB. S. mutans strains UA159 and △*fruB* were cultured in TV medium supplemented with 0.5% of inulin till mid-exponential phase. After centrifugation, the culture supernates were precipitated with trichloroacetic acid, washed and resuspended in 0.1 N NaOH. Bacterial cells were enzymatically broken up and fractionated into 2 samples, cell-wall-associated proteins (C.W.A.P) and cell membrane and cytoplasm (Mem/cyto). All 3 fractions were normalized to represent similar numbers of cells from each strain. After resolution of the protein samples on an SDS-PAGE, presence of FruB protein was visualized via Western blotting using affinity-purified anti-FruB antiserum.

Computer analysis of FruB sequence predicted the presence of a signal peptide spanning the first 24 amino acids, thus suggesting localization of the mature protein to the extracellular environment. A recombinant His-tagged FruB protein (rFruB) was engineered and overexpressed in E. coli, and the affinity purified protein was used to raise an anti-FruB antiserum, as detailed in the Methods section. Cell and supernatant fractions of wild-type UA159 and the Δ*fruB* strain were subjected to immuno-blotting using the anti-FruB antiserum. The results showed that FruB was detected only in culture supernates. Although the antisera also detected components in fractions from the cell envelope and cytoplasm, these proteins were not consistent with the predicted size of FruB and no difference in their intensity was noted between the wild type and Δ*fruB* (Fig. 2).

### FruB binds to and hydrolyzes levan.

Since, FruB has domains and catalytic residues that are conserved in other fructosidase enzymes (4), we hypothesized that enhancement by FruB of growth on levan was most likely due to its ability to bind and hydrolyze levans, potentially by interacting or cooperating with FruA to enhance hydrolysis of the polymers. When the Δ*fruB* strain was grown in TV-levan to which 100 nM purified rFruB was added, the growth delay typically observed for the *ΔfruB* strain was eliminated, with cultures displaying a lag phase similar in duration to the wild type. The addition of rFruB also partially rescued the growth of the Δ*fruA* and Δ*fruAB* strains, although it had a particularly notable effect on the latter (Fig. 1C).

**FIG 3:**
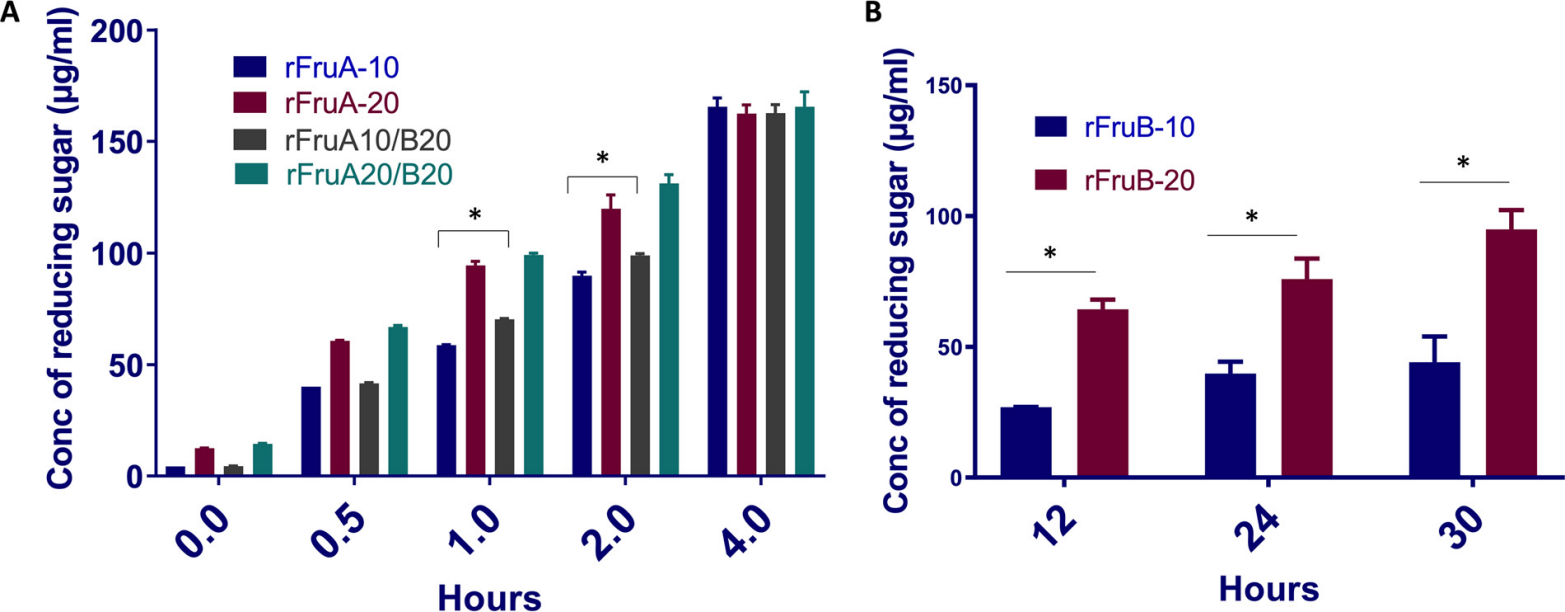
Hydrolysis of levan by recombinant rFruA and rFruB. (A) Hydrolysis of levan by recombinant rFruA and a mixture of rFruA/rFruB at different time points (0, 0.5, 1, 2 and 4 h). (B) Concentration of reducing sugar (μg/mL) was measured after hydrolysis of levan by 10 and 20 pmol of purified recombinant rFruB after 12, 24, and 30 h. Data are representative of three independent experiments performed at least in triplicate. (*P* < 0.05).

The ability of FruB to degrade and hydrolyze levan was assessed by measuring reducing sugar release from purified levan (20). Considering the phenotype of the fructanase mutants and complementation by the recombinant protein, we expected low to moderate levels of activities from rFruB *in vitro*. Levan was treated with 10 pmol or 20 pmol of rFruB and samples were collected and assayed for released reducing sugar at 12, 24 and 30 h. FruB hydrolyzed levan in a protein concentration-dependent manner, albeit at a much slower rate than FruA (Fig. 3A). The hypothesis that FruB could enhance the levan hydrolase activity of FruA was also tested. When FruA (10 and 20 pmol) and FruB (20 pmol) were combined in the reactions, the rate of hydrolysis was slightly faster under certain conditions compared to FruA alone (Fig. 3B).

### FruB functions as an endolevanase.

**FIG 4:**
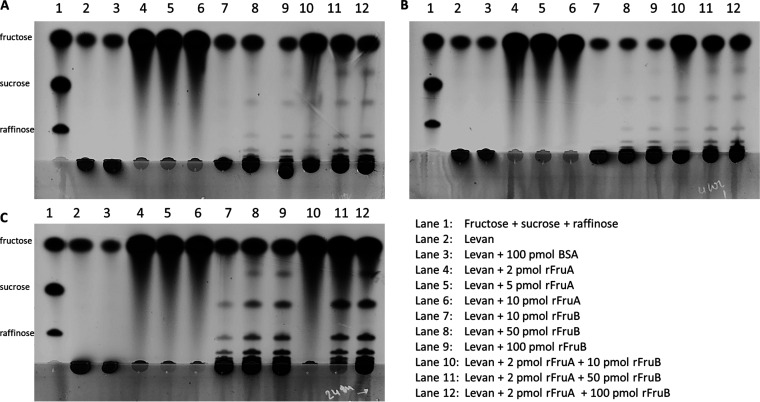
Thin Layer Chromatography (TLC) analysis of levan degradation. TLC at (A) 1 h, (B) 4 h and (C) 24 h time points. Lane 1 included a standard of fructose, sucrose and rafiinose; lane 2 0.5% untreated levan; lane 3 0.5% levan treated with BSA; lanes 4, 5, and 6 are levan treated with 2, 5, and 10 pmol of rFruA respectively; lanes 7, 8, and 9 were 0.5% levan treated with 10, 50, and 100 pmol of rFruB; and lanes 10, 11, and 12 were levan treated with a mixture of rFruA (2 pmol) with increasing concentrations of rFruB (10, 50 and 100 pmol). Data presented are from the same experiment and representative of multiple repeats across different days.

Measurements of reducing sugar does not provide definitive information about whether of FruB acts endo- or exo-hydrolytically on levan, so Thin Layer Chromatography (TLC) was performed to visualize products of FruB acting on levan. Consistent with a previous report (7), FruA degraded levan to completion, releasing only monosaccharides (fructose) in a time- and concentration-dependent manner (Fig. 4, lanes 4, 5, 6). No significant increase in free fructose was noted when rFruB alone was used to treat levan. Instead, when 100 pmol of rFruB was incubated for 24 h with levan, FruB liberated oligosaccharides, consistent with the enzyme functioning as an endolevanase (Fig. 4, lanes 7, 8, 9). When FruB was used in conjunction with 2 pmol FruA, we continued to observe fructose oligomers. Interestingly, the presence of FruB appeared to slow the release of free fructose by FruA, especially when used at high concentrations (Fig. 4, lanes 11, 12). This effect was made clear by both the reduction in fructose signals and the increased intensity in the proportion of incompletely digested levan.

### Analysis of *fruAB* operon transcripts.

**FIG 5:**
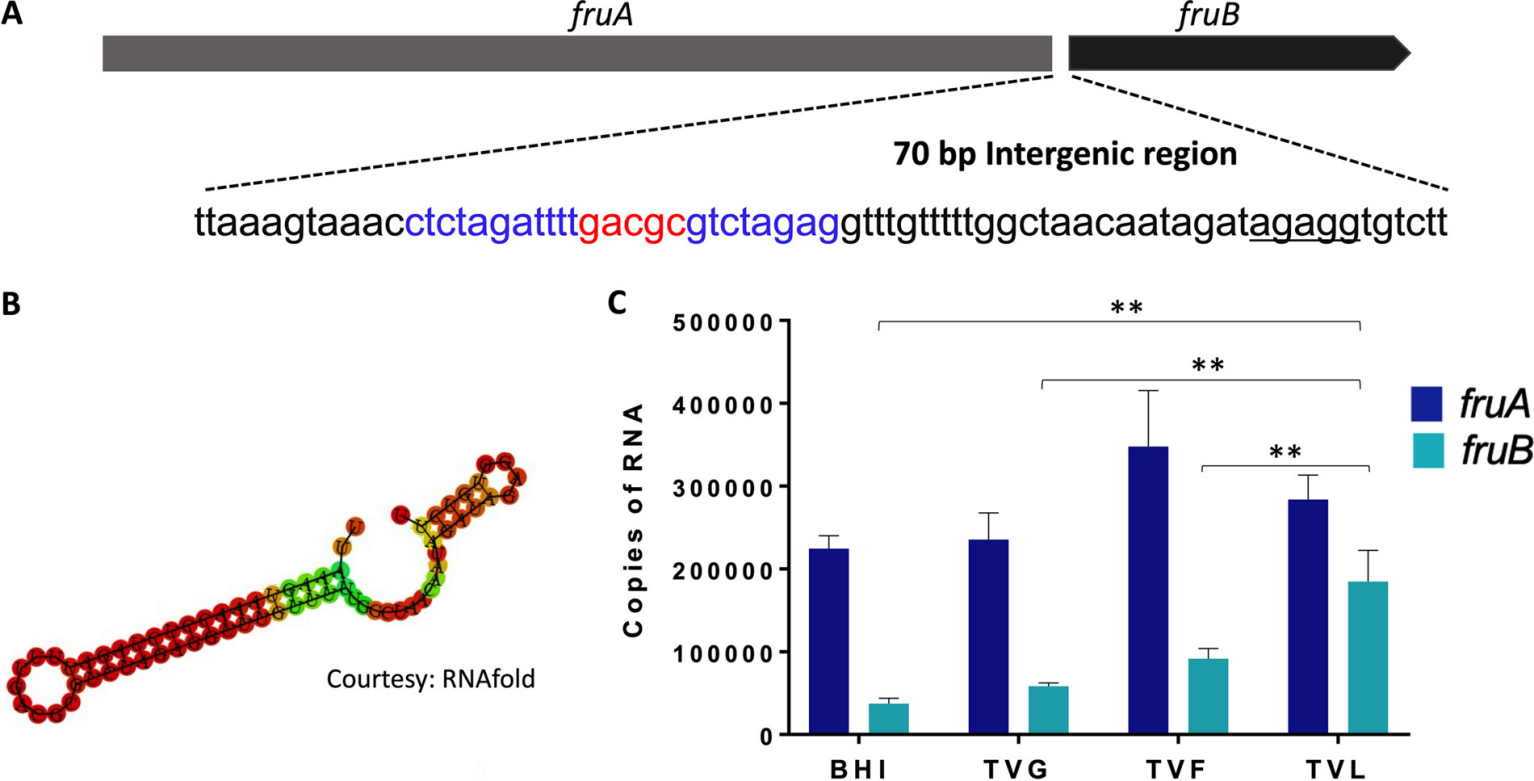
Transcription analysis of the *fruAB* operon. (A) genes *fruA* and *fruB* are separated by a 70-bp intergenic region (*igr*) that included a partial inverse repeat highlighted in blue. (B) The secondary structure of the *igr* was predicted using the RNAfold web browser (34), with a free energy [ΔG = -21.24 Kcal/mol]. (C) mRNA levels of *fruA* and *fruB* of S. mutans UA159 grown under different carbohydrate sources were measured by RT-qPCR. Data are representative of at least three independent biological replicates performed in triplicates. Values are average and error bars indicate standard deviation across replicates. *P* < 0.005, by Student's *t* test.

The *fruB* structural gene is 1,560 bp in length and is the second gene in an apparent two-gene operon preceded by *fruA* (Fig. 5A). A 70-bp intergenic region (*igr*) separates the stop codon of *fruA* from the start codon of *fruB.* Within this *igr* a stable stem-loop (SL) structure was predicted by RNAfold [ΔG= -21.24 Kcal/mol] (Fig. 5B). An apparent ribosome binding site (RBS, AGAGG) is located just upstream of *fruB*, but there did not appear to be sequences with similarity to canonical promoter sequence within the *igr*, as assessed by scanning of the region manually and using the online promoter finder tool BPROM (Softberry). As reported earlier, *fruA* and *fruB* can be co-transcribed (12). To measure the transcript levels of these genes under different growth conditions, qRT-PCR was performed. The relative expression levels of *fruB* and *fruA* varied as a function of the primary growth carbohydrate (Fig. 5C). Specifically, *fruB* was expressed at significantly lower levels than *fruA* in BHI, in TV medium with glucose (TVG) or in TV-fructose (TVF) (*P* value < 0.05); providing support for the idea that the *igr* may interfere with transcription read-through. Conversely, *fruB* expression increased substantially when the cells were grown in TV medium with levan as the sole supplemental carbohydrate (*P < *0.005) (Fig. 5C). We hypothesized that the higher expression levels of *fruB* in cells growing on levan could be attributable both to enhanced expression from the *fruA* promoter and destabilization of the stem-loop structure in the *igr*; although additional factors such as altered mRNA stability or post-transcriptional mRNA processing cannot be excluded.

**FIG 6:**
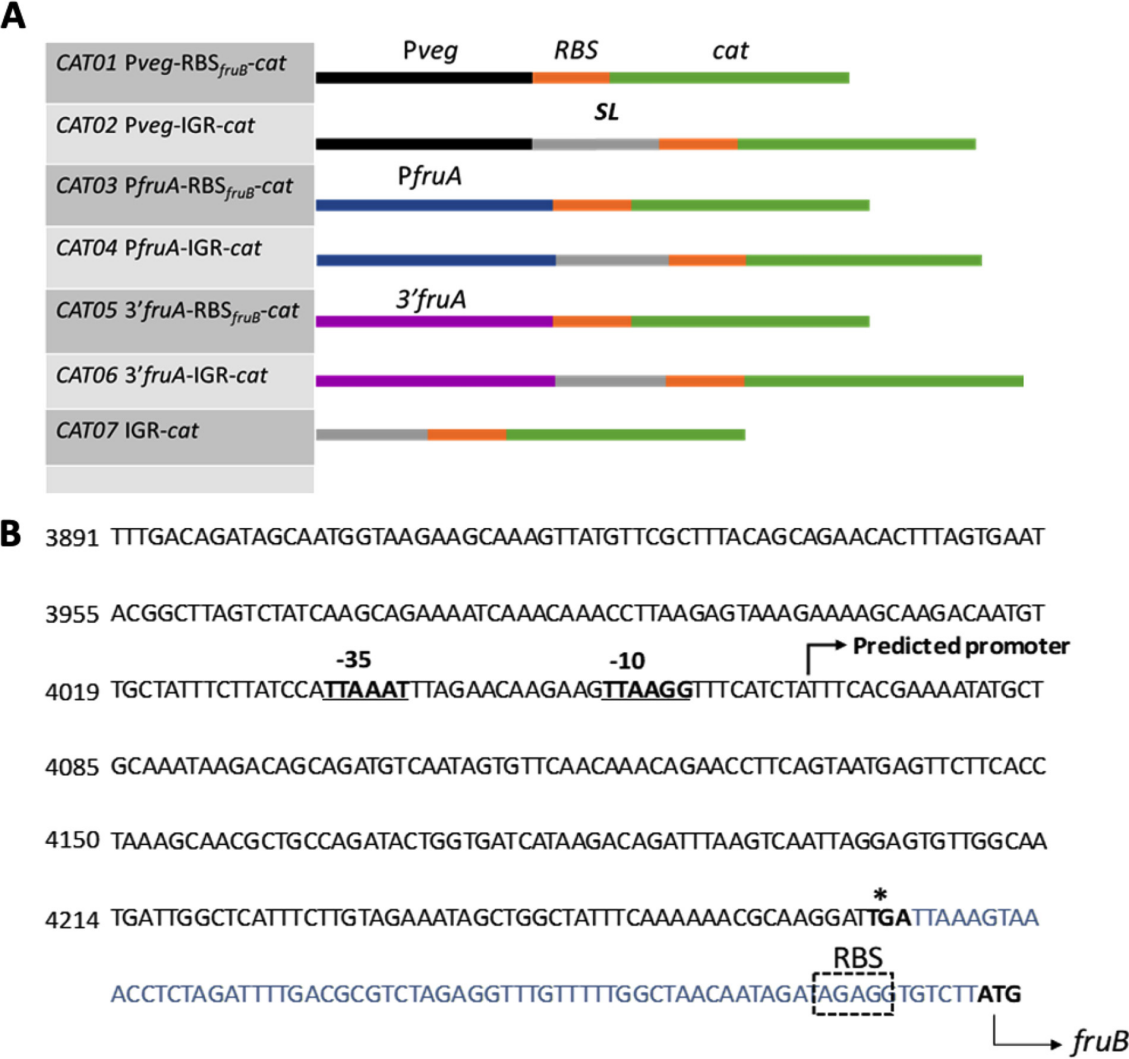
Construction of reporter gene fusions. (A) Schematic diagrams of the *cat*-fusion constructs used in this study. The black bars represent promoters of *P_veg_* (used as a control), the blue bars represent the *fruA* promoter region and the purple bars represent the 3′ end of the *fruA* coding sequence (B), presumably containing an internal promoter. The orange bars represent the putative ribosome binging site (RBS) of *fruB* gene, and the gray bars represent the rest of the 70-bp *igr* including a putative stem-loop structure (SL). The green bars represent the *cat* gene. (B) Relevant nucleotide sequence and likely features near the 3′ end of *fruA*. Online promoter prediction tool BPROM (SoftBerry) was used to assess the presence of a promoter. Underlined and in boldface are the extended -35 and -10 elements that may alternatively drive *fruB* transcription. The *igr* between *fruA* and *fruB* is highlighted in blue and the *fruB* RBS is represented in a box.

To examine whether the *igr* played a role in the regulation *fruB*, gene fusions were generated that contained various portions of the *igr* fused to a chloramphenicol acetyltransferase (CAT) reporter gene that lacked its own promoter and ribosome binding site (RBS). Schematics of the seven *cat* constructs (CAT01–CAT07) are provided in Fig. 6A. The *igr* is comprised of a stable stem-loop (SL) structure followed by an RBS-like sequence. The full length *igr* (70 bp) with the RBS (CAT02, 04, 06, 07) or a 13-bp sequence containing only the RBS (nt 58–70 of *igr*, CAT01, 03, 05) was fused to the *cat* gene, such that translation of the *cat* gene would always be driven by the cognate *fruB* RBS. The *fruA* promoter (*P_fruA_*), along with a 382-bp, 3′ region of the *fruA* gene, potentially possessing a promoter, was cloned upstream of the *igr*/RBS to drive expression of the *cat* translational fusions. *P_veg_* of Bacillus subtilis was used as a constitutive promoter to study the role of the suspected terminator in the *igr*. The constructs were integrated into the S. mutans strain UA159 genome in single copy at a site distant from the *fruAB* operon, as described in the methods section and elsewhere (21).

In cells grown in TVG or TV medium formulated with inulin (TVI), no significant CAT activity was observed when expression was driven by *P_fruA_* or the fragment containing the 3′ region of *fruA* upstream of the *igr* (CAT03, CAT04, CAT05, CAT06; Table 2). When cells were grown in TVF medium, substantial CAT activities were observed in the *P_fruA_*-driven construct containing only the *fruB* RBS (CAT03), but lower levels of CAT activity were noted when the entire *igr* was present (CAT04), indicating that the SL element in the intact *igr* may modulate the expression of *fruB* by decreasing transcriptional read-through. The most interesting behavior was observed when the cells were cultured in TV medium with 0.2% levan (TVL). Specifically, CAT activities by constructs CAT03 and CAT04 in TVL were significantly higher than any other conditions in which expression was driven by *P_fruA_*, although the presence of the SL element again significantly reduced CAT activity. Constructs containing the 3′ end of *fruA*, CAT05, in particular, exhibited measurable CAT activities when cells were grown with levan, indicative of the presence of a secondary promoter that may drive *fruB* expression and is inducible by levan. Similar to fusions made with *P_fruA_*, the CAT activities from this apparent secondary promoter were lower when an intact *igr* was present (CAT06, *p* < 0.05). The use of *P_veg_* as a promoter control and its weakened activity when the *igr* was present adds further support to the hypothesis that the SL element may act as a transcriptional terminator. Also of note, the *P_veg_*-IGR-*cat* fusion did not show enhanced expression when cells were growing on levan, compared to other carbohydrates. Collectively, the results provided support for the hypothesis that, in addition to the *fruA* promoter, a levan-responsive promoter within the 3′ end of *fruA* may contribute to *fruB* transcription, and that the *igr* can modulate *fruB* mRNA production, likely by attenuating transcriptional read-through.

**TABLE 2:** CAT activities of constructs with *igr* derivatives

Avg (SD)^*a*^ CAT sp act [nmol (mg protein)^−1^ min^−1^							
Media	*P_veg_*-RBS*_fruB_*-*cat*	*P_veg_*-IGR-*cat*	*P_fruA_*-RBS*_fruB_*-*cat*	*P_fruA_*-IGR-*cat*	3’*fruA*-RBS*_fruB_*-*cat*	3’*fruA*-IGR-*cat*	IGR-*cat*
TVG	50.21 (0.52)	4.55 (0.41)	7.96 (0.49)	0 (0)	0.42 (0.0)	0 (0)	0 (0)
TVF	45.46 (0.87)	3.08 (0.67)	14.46 (0.29)^*b*^	0.86 (0.0)	1.90 (0.39)^*b*^	0.32 (0.02)	0.75 (0.0)
TVL	77.43 (0.52)	5.47 (0.18)	72.84 (0.36)^*b*^	4.58 (0.26)^*b*^	3.10 (0.15)^*b*^	0.20 (0.12)	0.80 (0.42)
TVI	37.99 (0.80)	0 (0)	8.98 (0.45)	0.98 (0.25)	0.85 (0.18)	0 (0)	0 (0)

a ± standard deviation.

b Indicates statistical significance (*P* < 0.01, One-Way Anova) relative to cells grown using TVG medium.

### Loss of FruB affects the transcriptome in a carbohydrate-dependent manner.

**FIG 7:**
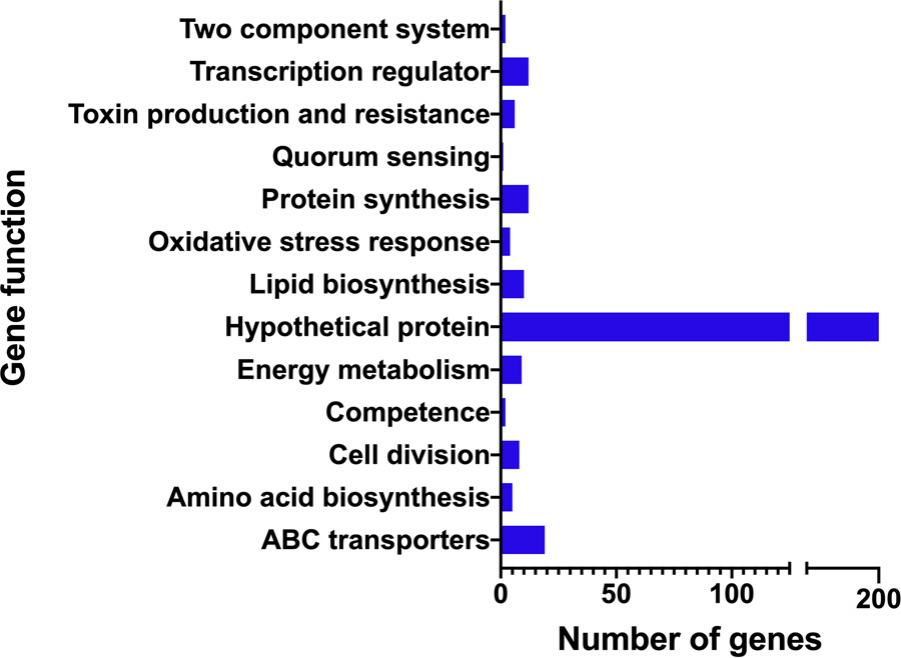
Cumulative analysis of transcriptomic data. RNA-seq data were obtained from WT UA159 and strain △*fruB*, grown in the presence of 0.2% levan as sole carbohydrate source. The bar diagram shows the numbers of genes with significant changes related to various cellular functions, collectively indicating a global change in the gene expression profile in *ΔfruB* as it adapts to the presence of levan.

The results presented above support that *fruB* expression is responsive to growth on levan and that growth of S. mutans on levan can be improved by FruB. To obtain a more comprehensive assessment of the role of FruB in S. mutans, WT and the *fruB* mutant were grown in TV medium with 0.2% levan as the sole carbohydrate source and were harvested at exponential phase (OD = 0.5). cDNA libraries were prepared from enriched mRNA and RNA-Seq was performed. Global transcriptome analysis revealed significant changes (FDR < 0.01, and fold of change > 2) in the expression of a total of 325 genes (77 downregulated, 248 upregulated), representing an array of cellular functions, including carbohydrate metabolism (e.g., SMU_99, SMU_102-104), cell division (e.g., *divIB*, *divIVA*, SMU_13, SMU_1513, SMU_1713), competence (e.g., *comB*, *comYC*, SMU_1903 to SMU_1913c), two component systems (e.g., *ciaRH*, *vicRK*, *covR*), oxidative stress responses (e.g., *dpr*, *sodA*, SMU_694), manganese and iron transport (e.g., SMU_182-185, SMU 1927-28) and quorum sensing (*luxS*), among others (Fig. 7, Table S1 for details). A large portion of the transcriptome affected by *fruB* deletion is comprised of hypothetical proteins or genes of unknown functions.

## DISCUSSION

The primary objective of this study was to explore the role of FruB in the metabolism of exopolysaccharide fructans that influence oral microbial ecology and oral biofilm virulence. Although FruA functions as the dominant fructan hydrolase in S. mutans, targeting inulins and levans, our research reveals a significant role for FruB in the utilization of levan for growth. That FruB plays a significant role in levan metabolism was further supported by the finding that *fruB* expression was highest in cells growing on levan, but not in cells growing on inulin or hexoses such as glucose or fructose. And, as noted above, the high degree of conservation of *fruB* in nearly all sequenced isolates of S. mutans is indicative of the potential for substantial selective pressure to maintain FruB activity in the human oral cavity. Also noteworthy is the apparent high degree of specificity of FruB for β2,6-linked fructans (levans), since the fructans produced by S. mutans are almost uniquely β2,1-linked (inulins). Given that FruA also has a strong preference of levans over inulins, it is reasonable to posit that the FruA and FruB fructanases of S. mutans evolved, and possibly co-evolved, mainly to scavenge fructans produced by S. salivarius and Actinomyces spp., while FruA allows for catabolism of the inulin-type fructans produced by S. mutans and many commensal oral streptococci.

The substrate used in this study was levan that was synthesized *in vitro* using a protein preparation containing the FTF enzyme from S. salivarius. Based on prior analysis of the structure and size of the products of the S. salivarius FTF (22), the fructans thus synthesized should be composed primarily of β (2, 6)-linked chains of fructose polymers with substantial branching with β(1, 2) linkages. FruA is a β-d-fructosidase that releases fructose exo-hydrolytically from the reducing ends of the fructan polymers. *In vitro* hydrolysis assay using rFruB and levan showed negligible release of free fructose after 4 h of incubation, although extended incubation for up to 24 h did show accumulation of free fructose. However, the dominant products released from the levan by rFruB at earlier time points were oligosaccharides. This pattern is not uncommon for polysaccharide endo-hydrolases and most consistent with FruB being an endolevanase. It is also possible that FruB could have some debranching activity. However, FruA can degrade levans and inulins to completion with no evidence of residual di-, tri- or oligosaccharides, so FruB need not have any other functions beyond endolevanase activity for S. mutans to completely utilize the fructans that are produced by those species that constitute the majority of the oral flora. Also implicit to these findings was the conclusion that rFruB binds efficiently to the levan molecules. In fact, rFruB at high concentrations hindered the activity of rFruA in releasing free fructose, perhaps due to competition for target sites on the substrate and the apparent lower efficiency of FruB in cleaving the polysaccharide. While our results demonstrate a function for FruB and, in all likelihood FruA and FruB cooperate to optimize levan utilization, much remains to be learned about how the amounts of FruA and FruB are modulated and how the enzymes function in the complex environments encountered in human oral biofilms.

It is perhaps reasonable to suggest the recombinant rFruB protein showed relatively low specific activities in our levan hydrolysis assays. We came to this conclusion on the basis of combined knowledge of naturally low FruB abundance in UA159 cultures (via semi-quantitative Western blotting; Fig. S2 in the supplemental material), the significant phenotype of the *fruB* mutant growing on levan, and the relatively high concentrations of rFruB needed to demonstrate biological activities *in vitro*. The exact cause of this discordance is not yet clear. However there are a few possible explanations. First, the rFruB protein was overproduced in a heterogeneous host E. coli as a fusion protein (with a 6× His tag) without its natural signal peptide, consequently lacking any potential post-translational modification (e.g., glycosylation), localization to specific cellular compartments/organelles, or complexing with partner protein(s). The last is especially relevant as the rFruA protein was produced in the same fashion as that of rFruB. As such, any requirement for post-translational processing of either protein by factors produced by S. mutans would have resulted in significant reduction in biological activities in our *in vitro* assays. FruA is a conditional cell envelope protein that depends on sortase and proper pH for attachment to peptidoglycan of the cell wall (14), so it can be found both extracellularly and on the cell surface depending on the conditions (e.g., pH). Previous research of a C-terminally Flag-tagged recombinant FruB protein expressed in UA159 showed localization of the fusion protein to the cell envelope (Fig. S3). Therefore, we are not ruling out the possibility that FruB could also be cell associated under certain conditions. Second, as mentioned above, a certain stoichiometry is likely required for these two gene products of the same operon to function properly. By Western blotting, FruA appears to be produced in *in vitro* culture in much greater abundance than FruB (Fig. S2), which is consistent with the apparent transcription attenuation that dampens *fruB* mRNA levels. Ample examples exist in nature where complexes of heterogeneous proteins are required for degradation of macromolecules including polysaccharides, e.g., the degradation of cellulose often requires multi-enzyme complexes (23). Further study of these two enzymes, perhaps by using samples prepared simultaneously from S. mutans cells or by employing live imaging technologies capable of differential labeling of both proteins, could help answer some of these questions. Lastly, other factors and/or conditions, such as pH, ionic strength, specific metal ions, are required for optimal fructan degradation. For simplicity, the buffers used in levan hydrolysis in this study was based on the preference of the FruA protein (7), including an acidic pH.

Prior evidence supported that *fruAB* could be co-transcribed from the *fruA* promoter, including RT-PCR, RNA-Seq tracings and analysis of mutants deficient in one or both genes (4, 8, 13). Although a computer analysis predicted the presence of a stem-loop secondary structure within the intergenic region (*igr*) between *fruA* and *fruB*, this study establishes directly that the *igr* can influence the expression of *fruB* under a variety of conditions. Though the exact mechanism(s) remains to be elucidated, transcription termination, alteration of mRNA stability or effects on translational efficiency are among the most likely. Notwithstanding, a significant discovery from this study was the identification of a functional promoter located within a 382-bp region in the 3′ portion of the *fruA* structural gene (Fig. 6B), and the *cat* gene expression driven by this promoter was enhanced in cells cultured with levan as the sole carbohydrate, compared to cells grown in glucose, fructose or inulin. We have characterized the transcription regulation of the *fruA* promoter with some detail, by identifying a four-component system (LevQRST) that activates its expression in response to fructose or mannose (24), and carbohydrate catabolite repression that reduces the transcription in response to preferred sugars. It remains to be clarified whether the LevQRST system is also responsible for controlling this secondary promoter. It should also be noted that the responsiveness of the *fruA* promoter (*P_fruA_*-RBS*_fruB_*-*cat*) measured here was much lower than our lab previously reported (8). However, in that study, *P_fruA_* activity was monitored using a *cat* fusion to the cognate *fruA* promoter, whereas in this study translation of *cat* expressed from the *fruA* promoter was dependent on the RBS of the *fruB* gene. Another paradigm system in regulation of catabolic genes is the *levA* gene in Bacillus subtilis, which has both transcriptional activation and antitermination as primary modes of regulation (25). Interestingly, the *fruA* mRNA in S. mutans has a large 5′ untranslated region (UTR) with a putative terminator, but antitermination does not seem to be a primary control point of *fruA* regulation (4). The possibility of the *fruAB igr* serving as a *cis-*acting element to respond to carbohydrate sources was tested in this study by inserting the *igr* behind the constitutive *P_veg_* promoter. Although cells carrying the *P_veg_* promoter construct (*P_veg_*-RBS*_fruB_*-*cat*) did show different levels of expression depending on the growth carbohydrate, *P_veg_*-IGR-*cat* did not show high activities in levan-grown cells. Thus, it does not appear that the primary role of the *igr* is to enhance the levels of *fruB* when cells encounter levan. Rather, the promoter nested within *fruA* appears to be the primary way in which cells can upregulate *fruB* when levans are present. Last, being capable of both activating the LevQRST circuit at low levels and inducing CCR at higher levels (24), there could exist a steady-state levels of fructose, perhaps at low mM concentrations, that is optimal for inducing *fruB* expression without triggering CCR. It is possible that the combined activities of FruA and FruB on levan, but not inulin, satisfy this requirement.

Finally, comparison of the transcriptomes of the wild-type and a *fruB* mutant growing on levan revealed a large collection of genes that show altered expression in cells lacking FruB. Changes in almost 200 hypothetical genes have indicated major physiological changes in a Δ*fruB* strains, most of which is not known at this point. Two notable regions of the genome with increased transcription in the *fruB* mutant were the genomic islands TnSmu1 (SMU_191 to SMU_223c) and TnSmu2 (SMU_1334 to SMU_1392c), the latter of which has been suggested to contribute to resistance against oxygen and H_2_O_2_ (26). Conversely, nearly every known gene with a proven role in oxidative stress (*dpr*, *sodA*, ferredoxin, SMU_540) was found to be downregulated in *ΔfruB* strain. Most of the genes linked to oxidative stress in S. mutans are under the control of global transcriptional regulator SpxA1 and SpxA2, which have been associated to the virulence potential of S. mutans (27–29). It is therefore assumed that a *ΔfruB* derivative could be less virulent than WT S. mutans under certain growth conditions, such as the presence of commensal oral bacteria that secrete hydrogen peroxides as survival mechanisms.

For survival several different bacteria employ a quorum sensing (QS) system, which interacts with various stimuli in the oral environment and functions as a bacterial intracellular signal transduction mechanism for controlling gene expression in response to population density (30, 31). The Com-dependent QS system of S. mutans that is known to be stimulated by CSP regulates critical bacterial functions such as bacteriocin production, genetic transformation and acid or stress tolerance. *ΔfruB* indicated upregulation in some of the *com* genes. *comB* (SMU_287), an accessory protein for the ComA ABC transporter which is required for the secretion of CSP was upregulated about 3-fold. Also upregulated in the *fruB* mutant was a cluster of genes, SMU_1903c to SMU_1913c, excluding SMU_1914 which encodes for mutacin V, located adjacent to the CSP-encoding *comC* gene and *comDE* two-component system. Recent studies of oral bacteria have provided evidence that another quorum sensing molecule significant in the physiology and survival of S. mtuans is AI-2 (autoinducer-2). It has been shown that *luxS* of S. mutans is involved in the regulation of stress tolerance and biofilm formation. Our lab has previously established a relationship between *luxS* and *fruA*; lack of *luxS* resulted in decreased *fruA* expression. However, no significant differences were observed with *gftB*, *gtfC*, *gbpB* and other genes related to biofilm formation (32). It was hypothesized that *luxS* regulates expression of *fruA* through perturbation of the expression of gene products involved in CCR. *fruA* and *fruB* are under the regulation of *fruA* promoter and therefore, *fruB* could also be regulated by CCR. Our RNA seq data revealed a significant reduction in *luxS* expression in *ΔfruB* compared to wild type UA159. Several of the genes showed similar expression pattern in *fruB* and *luxS* mutant strains. SMU_53, SMU_153, SMU_197c, SMU_422 and SMU_643 are all upregulated in both the mutant strains, clearly indicating f*ruB* and *luxS* may be regulated via the same feedback loop (33).

While we continue trying to understand the significance of these changes in gene expression, there are a few effects to consider. First, when S. mutans is grown on fructose, rather than glucose or galactose, the cells display a substantially altered transcriptome; interestingly many of the genes affected are involved in competence or stress tolerance. Growth on fructose generates certain metabolites that are likely absent or present in substantially lower concentrations in cells grown on other hexoses, e.g., F-1-P and F-6-P, respectively, that can have substantial effects on gene regulation (34, 35). A reduction in the efficiency of release of fructose from levans and associated slower internalization of fructose, along with the rate of carbohydrate flow through catabolic pathways (36), could thus alter expression of fructose-responsive genes and potentially central metabolism. Second, the production of a variety of small fructose polymers (oligolevans) by the activities of FruB on levan could influence gene regulation by engaging certain carbohydrate-binding proteins or pathways required for transporting and catabolizing these carbohydrates (e.g., ABC-type transporters and PTS enzymes).

### Concluding remarks.

This study represents the first significant breakthrough in understanding the function of FruB in fructan metabolism. The ability to synthesize then utilize storage polysaccharides allows oral biofilms to optimize the capture of transiently provided dietary carbohydrates. Based on data presented here, the highly conserved S. mutans *fruB* gene should enhance competition of S. mutans for the carbon and energy stored in the levans that were produced by competing oral bacteria enzyme, giving S. mutans a distinct ecological advantage under conditions conducive to the initiation and progression of dental caries. Further analysis of the biochemistry of fructan utilization and how it is controlled in polymicrobial communities in the human oral cavity could lead to the development of novel approaches to diminish the cariogenic potential of oral biofilms.

## MATERIALS AND METHODS

### Bacterial strains and growth conditions.

Strains used in this study are listed in Table 1. S. mutans UA159 and its derivatives were routinely cultured in brain heart infusion (BHI; Difco Laboratories, MI) broth and maintained on BHI agar in a 5% CO_2_ aerobic atmosphere at 37°C. When needed, kanamycin (1 mg mL^−1^) or spectinomycin (1 mg mL^−1^) was added to broth or BHI agar. Growth assays for S. mutans were performed in TV-base medium (12) formulated with various carbohydrate sources at the indicated concentrations.

Cells for chloramphenicol acetyltransferase (CAT) assays and for the extraction of RNA were obtained by growing S. mutans in TV medium supplemented with various carbohydrates (10 mM each glucose and fructose and 0.2% of levan and inulin) until mid-exponential phase (OD_600_ = 0.5). Cells were then harvested by centrifugation (3400 × *g*) and cell pellets were frozen at −80°C until CAT assays or RNA extraction was performed.

For monitoring growth of S. mutans strains, cells were cultured in BHI broth overnight, diluted into fresh BHI (1:25), incubated until cells reached mid-exponential phase (OD_600_ = 0.5), and then diluted 1:150 into TV medium containing the specified carbohydrates. The OD_600_ of the bacterial cultures was recorded using a Bioscreen-C monitor (Oy Growth Curves AB, Helsinki, Finland) maintained at 37°C, with 60 μL of mineral oil added to each well to mitigate the inhibitory effects of oxygen on growth of S. mutans.

### DNA manipulation and mutant strain construction.

Standard techniques were applied for preparation and manipulation of DNA (13). All DNA restriction and modifying enzymes were purchased from New England BioLabs (Beverly, MA) and used as recommended by the supplier. Oligonucleotides for PCR amplifications were custom synthesized by Integrated DNA Technologies, Inc. (Coralville, IA) and are described in Table 3. Multiple mutagenesis steps were carried out to construct various S. mutans strains, using allelic exchange with non-polar elements encoding resistance to kanamycin (km) or spectinomycin (sp) to replace the genes of interest without disrupting downstream gene expression. Genetically competent cells were prepared by growth in the presence of 100 nM competence-stimulating peptide (CSP) in BHI medium (3). To introduce a polar mutation into the *fruAB* operon, an Ω kanamycin cassette (37) was used. The configuration of the double crossover integration of the antibiotic resistance markers were confirmed by PCR amplification coupled with Sanger sequencing.

**TABLE 3:** Primers used in this study

Primers	Sequences (5′–3′)
fruA-1000 up-S	TTATTATTGGACAAGCAGCGCTGGATAG
fruA-1000 up-AS	TTCCTCTTTTTCCTTGCTCTCCTTTTCAAATTTATGAAAC
NpKan-fruA-S	AGCAAGGAAAAAGAGGAAGGAAATAATAAATGGC
NpKan-fruA-AS	GTTTACTTTAACTAAAACAATTCATCCAGTAAAATATAATATTTTATTTTC
fruA-1000 down-S	ATTGTTTTAGTTAAAGTAAACCTCTAGATTTTGACXG
fruA-1000 down-AS	CATCCAAGCTATACTCTTAACGACG
fruB-1000 up-S	GATCAATACCACCATATTAAAGTCAC
fruB-1000 up-AS	TTCCTCTTTAAGACACCTCTATCTATTGTTAGCC
NpKan-fruB-S	AGGTGTCTTAAAGAGGAAGGAAATAATAAATGGC
NpKan-fruB-AS	TTATTGCTGCTAAAACAATTCATCCAGTAAAATATAATATTTTATTTTC
fruB-1000 down-S	ATTGTTTTAGCAGCAATAAAACTAAGTCTTAAGTCG
fruB-1000 down-AS	TATCAGCAACTTGAACTCGATGAAG
fruBBamH1pIB184-S	CGCGGATCCTCTAGATTTAAGGAGATATACATATGCTAAAGAAAAAATTAG
fruBXho1pIB184-AS	CCGCTCGAGTTACGTTTCCAAATTAGC
fruA-RT_S	CGACTAGTACGGATTTGATTCACTGG
fruA-RT-AS	CCACCCTTAGCCGTCTTAAATAAGC
fruB-RT_S	AGGTTAATGATGTACCTAGCTGAGGG
fruB-RT-AS	CCACAAGACCTAAGTCTTTTCCCC
luxS-RT_S	ACTGTTCCCCTTTTGGCTGTC
luxS-RT-AS	AACTTGCTTTGATGACTGTGGC
divIB-RT_S	AATGGTACCCGTGTTGATACC
divIB-RT-AS	GAGGTTCGAGAACTGGTTCAA
dpr-RT_S	GTGGTTCAGGCTTCCTTTATCT
dpr-RT-AS	TTGATTACTATCGGTGGCGC
gyrA-RT_S	CCAAGAATCTGCTGTCCG
gyrA-RT-AS	TTGCGACTATCTGCTATGTG

### Construction of reporter strains.

Double-stranded gene fragments containing the *P_veg_* promoter from Bacillus subtilis, the *fruA* gene promoter (*P_fruA_*), and the regions 5′ to the *fruB* coding sequence including the promoter and the ribosome-binding site (RBS) from S. mutans UA159 were synthesized using gBlocks by Integrated DNA Technologies (Coralville, IA, USA). The DNA fragments were further digested with restriction enzymes, ligated into integration vector pJL84 harboring a staphylococcal chloramphenicol acetyltransferase (CAT) gene lacking its intrinsic promoter and RBS and introduced as a single copy into the *mtlA-phnA* region of the chromosome of S. mutans UA159 (20). PCR was used to screen the transformants and DNA sequencing was used to verify the integrity of the inserted reporter gene constructs (38).

### Purification of recombinant proteins, fractionation, and Western blotting.

Both FruA and FruB have predicted N-terminal leader sequences that would facilitate secretion of the proteins. The recombinant proteins used in this study were constructed such that the signal peptides were omitted, but the remainder of the proteins were intact. Expression vector pQE30 (Qiagen, Heidelberg, Germany), which allows for positioning of a His-tag at the N-terminus via multiple cloning sites, was used to facilitate protein expression in Escherichia coli. The *fruA* (121- 4272 nt) and *fruB* (76-1560 nt) genes were amplified using a set of primers (Table 2) and then digested with restriction enzymes (BamHI and HindIII) for directional cloning in pQE30, followed by transformation into E. coli M15 (39). The resulting constructs were confirmed by DNA sequencing. The expression of each recombinant protein was induced by adding isopropyl-thio-β-d-galactopyranoside (IPTG) to a final concentration of 0.02 mM to mid-exponential phase cells. Purification of the recombinant proteins, designated rFruA and rFruB, were carried out using nickel-nitrilotriacetic (Ni-NTA)-agarose (Qiagen) according to supplier’s instructions, with minor modifications. To improve the solubility and stability of the recombinant proteins, 10% glycerol, EDTA-free protease inhibitor cocktail (cOmplete, Roche, Basel, Switzerland) and 500 μM phenylmethylsulfonyl fluoride (PMSF) (Sigma, St. Louis, MO) were added in the recommended buffer.

The purified rFruB protein was used to raise rabbit polyclonal antiserum (Lampire Biological Laboratories, Pipersville, PA). Anti-FruB antiserum was subsequently affinity-purified against immobilized rFruB antigen before use in immuno-blotting. Cell fractionation was carried out according to a protocol previously developed for S. mutans, with modifications detailed elsewhere (40).

### Synthesis and purification of levan.

Levan was synthesized using raffinose as a substrate and supernates prepared from Streptococcus salivarius 57.1, which produces a fructosyltransferase (FTF) enzyme that makes predominantly β2,6-linked fructan. Briefly, cells from a 200 mL overnight culture of *S. salivarius* 57.1 grown in BHI were harvested and washed in sterile deionized water. Cells were further resuspended in 10 mL of 20 mM potassium phosphate buffer (pH 6.5) containing 1 mM calcium chloride, 1 mM sodium azide and 0.5% raffinose (Sigma, St. Louis, MO) and incubated at 37°C for an hour to induce secretion of FTF. Cells were harvested, the supernatant fluid was filtered through a 0.25-μm membrane filter (Millipore, Burlington, MA) and the liquid was transferred into a dialysis tube with a molecular weight cutoff 6–8 kDa (kDa). At the same time, raffinose was dissolved with mild heating and gentle agitation in 200 mL of 20 mM potassium phosphate buffer (pH 6.5) to prepare a 10% (wt/vol) solution. The dialysis tube containing the FTF protein secreted by *S. salivarius* in the supernatant preparation was submerged in a beaker containing the 10% raffinose solution. Synthesis of levan, which accumulated in the dialysis bag, was carried out overnight at 37°C with gentle agitation. A white substance appeared in the dialysis tube after approximately 2 h of incubation at 37°C. The dialysis tube was rinsed with deionized water and then dialyzed twice for a total of 16 h against 1 L of sterile, deionized water at 4°C. Finally, levan was collected and stored at −20°C in aliquots. A small aliquot of levan was diluted 50-fold and subjected to acid hydrolysis in 0.5 M acetic acid at 70°C for 1 h. Total carbohydrate of the hydrolyzed levan preparation was quantified by measuring reducing sugar and used as estimates of the synthetic levan (20).

### Fructan hydrolase activity.

To assess the hydrolase activity of the recombinant proteins, the liberation of reducing sugar from purified levan was performed (20). To measure reducing sugar in samples, DNS reagent (1 mL) was added to 0.1 mL of sample and the mixtures were incubated at 95°C for 5 min. Known concentrations of fructose (0, 40, 80, 120, 160 and 200 μg/mL) were used to generate a standard curve each time an assay was performed. The absorbance was measured at 540 nm.

### Thin Layer Chromatography.

The degradation products of levan were analyzed by TLC following previously described protocols, with minor modifications (41, 42). Briefly, 0.5% (wt/vol) of levan was prepared with 10 mM 2-(*N*-morpholino) ethanesulfonic acid (MES) buffer (pH 6.0), and mixed with specified amounts of rFruA, rFruB, or a combination of both proteins, followed by incubation at 37°C for 1, 4, or 24 h. Immediately after, samples were placed on ice until being assayed by TLC. One μL of each sample was spotted onto an aluminum TLC Silica gel 60 F_254_ plate (Merck Millipore) and chromatographed twice with chloroform: acetic acid: water (60:70:10; vol/vol/vol). Reference carbohydrates were included as standards at the following concentrations: 30 mM fructose (monosaccharide), 30 mM sucrose (disaccharide), and 10 mM raffinose (trisaccharide). After drying of the TLC plates, spots containing fructose or fructose polymers were visualized using a urea spray (6 g urea, 32 mL water, 158 mL 2-butanol, 10 mL ethanol, and 11.2 mL orthophosphoric acid), followed by heating at 100°C in a fume hood for 1 h.

### CAT assay.

CAT (chloramphenicol acetyltransferase) activity (43) was measured in washed and snap-frozen cells stored at −80°C following an established protocol described elsewhere (3). CAT activity was expressed as the amount of lysate needed to acetylate 1 nmol of chloramphenicol × min^−1^ × (mg of protein)^−1^.

### RNA extraction and qRT-PCR.

Exponentially growing S. mutans (7 mL in BHI or TV medium supplemented with different carbohydrates) were harvested at OD_600_ = 0.5 and treated with bacterial RNAprotect (Qiagen, Germantown, MD) for 5 min. Cells were harvested and resuspended in 280 μL of TE (50/10, pH 8.0) with 0.4% SDS. Acidic phenol (300 μL, pH 4.3) and 0.25 mL of glass beads (avg. Diameter = 0.1 mm) were added, and the mixture was homogenized in a Bead-beater (Biospec Products, OK, USA) twice for 30 s at 4°C. The supernates were collected after centrifugation at 10,000 × *g* for 10 min at room temperature and the supplier’s (Qiagen) instructions were followed to isolate RNA. DNase treatment was performed on-column using RNase free DNase (Qiagen). Finally, total RNA was recovered in 40 μL of RNase free water. A Nanodrop spectrophotometer (Thermo Scientific, Wilmington, DE) was used to determine the purity and concentration of total RNA. The Superscript III kit (Invitrogen by Life Technologies, Carlsbad, CA) was used to synthesize first strand cDNA from 1 μg of total RNA using random hexamers according to a protocol from the supplier. Various mRNA transcripts were quantified by real-time PCR (RT-PCR) using gene-specific primers (Table 2) and data were normalized to housekeeping genes, as indicated. Quantitative real-time PCR (qRT-PCR) was carried out using an iCycler real-time PCR detection system (CFX96-Bio-Rad). Primers used in this study are listed in Table 2.

### Transcriptome analysis (RNA-seq).

Cells were grown in TV medium supplemented with 0.2% levan as the sole carbohydrate source. Total RNA was isolated and purified from S. mutans as described above. To remove 16S and 23S RNAs, 6 μg of high-quality total RNA was treated twice with the MICROBExpress Bacterial mRNA Enrichment Kit (Ambion of Life Technologies, Grand Island, NY, USA), then the RNA was precipitated with ethanol. The purified mRNA was resuspended in 25 μL of nuclease free water. The quality of enriched mRNA samples was analyzed using an Agilent Bioanalyzer (Agilent Technologies, Santa Clara, CA, USA). cDNA libraries were generated from 100 ng of the enriched mRNA samples using the NEBNext Ultra Directional RNA library prep kit for Illumina and NEBNext multiplex oligonucleotides for Illumina (New England BioLabs, Ipswich, MA). Deep sequencing was performed by the NextGen DNA sequencing Core Laboratory of Interdisciplinary Center for Biotechnology Research at the University of Florida (Gainesville, FL). A Galaxy server hosted by the high-performance research computing center at the University of Florida (HiPerGator 2.0) was used to map the reads. Treatment of the Illumina sequence data and statistical analyses were performed as described elsewhere (34, 44).

### Data availability.

Gene expression data have been deposited in the NCBI Gene Expression Omnibus (GEO) database (https://www.ncbi.nlm.nih.gov/geo/) under GEO Series accession number GSE200727.

## References

[1] Moye ZD, Zeng L, Burne RA. 2014. Fueling the caries process: carbohydrate metabolism and gene regulation by *Streptococcus mutans*. J Oral Microbiol 6:24878. doi:10.3402/jom.v6.24878. PMC4157138 25317251

[2] Zeng L, Chakraborty B, Farivar T, Burne RA. 2017. Coordinated regulation of the EII ^Man^ and *fruRKI* operons of *Streptococcus mutans* by global and fructose-specific pathways. Appl Environ Microbiol 83:e01403-17. 28821551 10.1128/AEM.01403-17 PMC5648919

[3] Chakraborty B, Burne RA. 2017. Effects of arginine on *Streptococcus mutans* growth, virulence gene expression, and stress tolerance. Appl Environ Microbiol 83:e00496-17. doi:10.1128/AEM.00496-17. 28526785 PMC5514675

[4] Wen ZT, Burne RA. 2002. Analysis of cis- and trans-acting factors involved in regulation of the *Streptococcus mutans* fructanase gene (fruA). J Bacteriol 184:126–133. doi:10.1128/JB.184.1.126-133.2002. 11741852 PMC134753

[5] Burne RA, Chen YYM, Wexler DL, Kuramitsu H, Bowen WH. 1996. Cariogenicity of *Streptococcus mutans* strains with defects in fructan metabolism assessed in a program-fed specific-pathogen-free rat model. J Dent Res 75:1572–1577. doi:10.1177/00220345960750080801. 8906125

[6] Birkhed D, Rosell K-G, Granath K. 1979. Structure of extracellular water-soluble polysaccharides synthesized from sucrose by oral strains of *Streptococcus mutans*, *Streptococcus salivarius*, *Streptococcus sanguis* and *Actinomyces viscosus*. Archs Oral Biol 24:53–61. doi:10.1016/0003-9969(79)90175-4. 292363

[7] Burne RA, Schilling K, Bowen WH, Yasbin RE. 1987. Expression, purification, and characterization of an Exo-β-D-fructosidase of *Streptococcus mutans*. J Bacteriol 169:4507–4517. doi:10.1128/jb.169.10.4507-4517.1987. 3308844 PMC213815

[8] Burne RA, Wen ZT, Chen YYM, Penders JEC. 1999. Regulation of expression of the fructan hydrolase gene of *Streptococcus mutans* GS-5 by induction and carbon catabolite repression. J Bacteriol 181:2863–2871. doi:10.1128/JB.181.9.2863-2871.1999. 10217779 PMC93730

[9] Ehrlich J, Stivala SS, Bahary WS, Garg SK, Long LW, Newbrun E. 1975. Levans: I. Fractionation, solution viscosity, and chemical analysis of levan produced by *Streptococcus salivarius*. J Dent Res 120:102–114. 1054339

[10] Gold W, Preston FB, Lache MC, Blechman H. 1974. Production of levan and dextran in plaque in vivo. J Dent Res 53:442–446. doi:10.1177/00220345740530024401. 4521906

[11] Higuchi M, Iwami Y, Yamada T, Araya S. 1970. Levan synthesis and accumulation by human dental plaque. Arch Oral Biol 15:563–567. doi:10.1016/0003-9969(70)90111-1. 5270188

[12] Zeng L, Wen ZT, Burne RA. 2006. A novel signal transduction system and feedback loop regulate fructan hydrolase gene expression in *Streptococcus mutans*. Mol Microbiol 62:187–200. doi:10.1111/j.1365-2958.2006.05359.x. 16987177

[13] Burne RA, Penders JEC. 1994. Differential localization of the *Streptococcus mutans* GS-5 fructan hydrolase enzyme, FruA. FEMS Microbiol Lett 121:243–249. doi:10.1111/j.1574-6968.1994.tb07105.x. 7926677

[14] Burne RA, Penders JEC. 1992. Characterization of the *Streptococcus mutans* GS-5 fruA gene encoding exo-β-D-fructosidase. Infect Immun 60:4621–4632. doi:10.1128/iai.60.11.4621-4632.1992. 1398976 PMC258211

[15] Ogawa A, Furukawa S, Fujita S, Mitobe J, Kawarai T, Narisawa N, Sekizuka T, Kuroda M, Ochiai K, Ogihara H, Kosono S, Yoneda S, Watanabe H, Morinaga Y, Uematsu H, Senpuku H. 2011. Inhibition of *Streptococcus mutans* biofilm formation by *Streptococcus salivarius* FruA. Appl Environ Microbiol 77:1572–1580. doi:10.1128/AEM.02066-10. 21239559 PMC3067281

[16] Tong H, Zeng L, Burne RA. 2011. The EIIAB man phosphotransferase system permease regulates carbohydrate catabolite repression in *Streptococcus gordonii*. Appl Environ Microbiol 77:1957–1965. doi:10.1128/AEM.02385-10. 21239541 PMC3067331

[17] Davies G, Henrissat B. 1995. Structures and mechanisms of glycosyl hydrolases. Structure 3:853–859. doi:10.1016/S0969-2126(01)00220-9. 8535779

[18] Wen ZT, Burne RA. 2001. Construction of a new integration vector for use in *Streptococcus mutans*. Plasmid 45:31–36. doi:10.1006/plas.2000.1498. 11319929

[19] Palmer SR, Burne RA. 2015. Post-transcriptional regulation by distal Shine-Dalgarno sequences in the grpE-dnaK intergenic region of *Streptococcus mutans*. Mol Microbiol 98:302–317. doi:10.1111/mmi.13122. 26172310 PMC4666293

[20] Viigand K, Visnapuu T, Mardo K, Aasamets A, Alamäe T, Alamäe T. 2016. Maltase protein of Ogataea (Hansenula) polymorpha is a counterpart to the resurrected ancestor protein ancMALS of yeast maltases and isomaltases. Yeast 33:415–432. doi:10.1002/yea.3157. 26919272 PMC5074314

[21] Simms PJ, Boyko WJ, Edwards JR. 1990. The structural analysis of a levan produced by *Streptococcus salivarius* SS2. Carbohydr Res 208:193–198. doi:10.1016/0008-6215(90)80099-o. 1964870

[22] Schwarz WH. 2001. The cellulosome and cellulose degradation by anaerobic bacteria. Appl Microbiol Biotechnol 56:634–649. doi:10.1007/s002530100710. 11601609

[23] Zeng L, Burne RA. 2008. Multiple sugar: phosphotransferase system permeases participate in catabolite modification of gene expression in *Streptococcus mutans*. Mol Microbiol 70:197–208. doi:10.1111/j.1365-2958.2008.06403.x. 18699864 PMC2583961

[24] Martin-Verstraete I, Stulke J, Klier A, Rapoport G. 1995. Two different mechanisms mediate catabolite repression of the *Bacillus subtilis* levanase operon. J Bacteriol 177:6919–6927. doi:10.1128/jb.177.23.6919-6927.1995. 7592486 PMC177561

[25] Wu C, Cichewicz R, Li Y, Liu J, Roe B, Ferretti J, Merritt J, Qi F. 2010. Genomic island TnSmu2 of *Streptococcus mutans* harbors a nonribosomal peptide synthetase-polyketide synthase gene cluster responsible for the biosynthesis of pigments involved in oxygen and H_2_O_2_ tolerance. Appl Environ Microbiol 76:5815–5826. doi:10.1128/AEM.03079-09. 20639370 PMC2935078

[26] Derr AM, Faustoferri RC, Betzenhauser MJ, Gonzalez K, Marquis RE, Quivey RG. 2012. Mutation of the NADH oxidase gene (Nox) reveals an overlap of the oxygen- and acid-mediated stress responses in *Streptococcus mutans*. Appl Environ Microbiol 78:1215–1227. doi:10.1128/AEM.06890-11. 22179247 PMC3273017

[27] Chen L, Ge X, Wang X, Patel JR, Xu P. 2012. SpxA1 involved in hydrogen peroxide production, stress tolerance and endocarditis virulence in *Streptococcus sanguinis*. PLoS One 7:e40034. doi:10.1371/journal.pone.0040034. 22768210 PMC3386922

[28] Galvão LCC, Miller JH, Kajfasz JK, Scott-Anne K, Freires IA, Franco GCN, Abranches J, Rosalen PL, Lemos JA. 2015. Transcriptional and phenotypic characterization of novel Spx-regulated genes in *Streptococcus mutans*. PLoS One 10:e0124969. doi:10.1371/journal.pone.0124969. 25905865 PMC4408037

[29] Cvitkovitch DG, Li YH, Ellen RP. 2003. Quorum sensing and biofilm formation in Streptococcal infections. J Clin Invest 112:1626–1632. doi:10.1172/JCI20430. 14660736 PMC281653

[30] Suntharalingam P, Cvitkovitch DG. 2005. Quorum sensing in Streptococcal biofilm formation. Trends Microbiol 13:3–6. doi:10.1016/j.tim.2004.11.009. 15639624

[31] Wen ZT, Burne RA. 2004. LuxS-mediated signaling in *Streptococcus mutans* is involved in regulation of acid and oxidative stress tolerance and biofilm formation. J Bacteriol 186:2682–2691. doi:10.1128/JB.186.9.2682-2691.2004. 15090509 PMC387784

[32] Sztajer H, Lemme A, Vilchez R, Schulz S, Geffers R, Yip CYY, Levesque CM, Cvitkovitch DG, Wagner-Döbler I. 2008. Autoinducer-2-regulated genes in *Streptococcus mutans* UA159 and global metabolic effect of the luxS mutation. J Bacteriol 190:401–415. doi:10.1128/JB.01086-07. 17981981 PMC2223724

[33] Zeng L, Burne RA, Robert BC. 2021. Molecular mechanisms controlling fructose-specific memory and catabolite repression in lactose metabolism by *Streptococcus mutans*. Mol Microbiol 115:70–83. doi:10.1111/mmi.14597. 32881130 PMC7854510

[34] Biswas I, Jha JK, Fromm N. 2008. Shuttle expression plasmids for genetic studies in *Streptococcus mutans*. Microbiology (Reading) 154:2275–2282. doi:10.1099/mic.0.2008/019265-0. 18667560 PMC4110107

[35] Moye ZD, Zeng L, Burne RA. 2014. Modification of gene expression and virulence traits in *Streptococcus mutans* in response to carbohydrate availability. Appl Environ Microbiol 80:972–985. doi:10.1128/AEM.03579-13. 24271168 PMC3911228

[36] Lorenz R, Bernhart SH, Höner Zu Siederdissen C, Tafer H, Flamm C, Stadler PF, Hofacker IL. 2011. ViennaRNA Package 2.0. Algorithms Mol Biol 6:26. doi:10.1186/1748-7188-6-26. 22115189 PMC3319429

[37] Seaton K, Ahn SJ, Sagstetter AM, Burne RA. 2011. A transcriptional regulator and ABC transporters link stress tolerance, (p)ppGpp, and genetic competence in *Streptococcus mutans*. J Bacteriol 193:862–874. doi:10.1128/JB.01257-10. 21148727 PMC3028664

[38] Park WJ, You SH, Choi HA, Chu YJ, Kim GJ. 2015. Over-expression of recombinant proteins with N-terminal His-tag via subcellular uneven distribution in *Escherichia coli*. Acta Biochim Biophys Sin (Shanghai) 47:488–495. doi:10.1093/abbs/gmv036. 25994007

[39] Kheir Z-H, Brady LJ. 2008. Isolation and solubilization of cellular membrane proteins from bacteria. Methods Mol Biol 425:287–293. 18369904 10.1007/978-1-60327-210-0_23

[40] Miller GL. 1959. Use of dinitrosalicylic acid reagent for determination of reducing sugar. Anal Chem 31:426–428. doi:10.1021/ac60147a030.

[41] Visnapuu T, Meldre A, Põšnograjeva K, Viigand K, Ernits K, Alamäe T. 2019. Characterization of a maltase from an early-diverged non-conventional yeast Blastobotrys adeninivorans. Int J Mol Sci 21:297. doi:10.3390/ijms21010297. PMC6981392 31906253

[42] Shaw WV. 1975. Chloramphenicol acetyltransferase from chloramphenicol-resistant bacteria. Methods Enzymol 43:737–755. doi:10.1016/0076-6879(75)43141-X. 1094240

[43] Zeng L, Choi SC, Danko CG, Siepel A, Stanhope MJ, Burne RA. 2013. Gene regulation by CcpA and catabolite repression explored by RNA-Seq in *Streptococcus mutans*. PLoS One 8:e60465. doi:10.1371/journal.pone.0060465. 23555977 PMC3610829

[44] Zeng L, Burne R.a. 2016. Sucrose- and fructose-specific effects on the transcriptome of *Streptococcus mutans* probed by RNA-Seq. Appl Environ Microbiol 82:146–115. doi:10.1128/AEM.02681-15. 26475108 PMC4702655

