# Supplementary material for: The fruB Gene of Streptococcus mutans Encodes an Endo-Levanase That Enhances Growth on Levan and Influences Global Gene Expression
Source: Microbiol Spectr. 2022 May 19;10(3):e00522-22. doi: 10.1128/spectrum.00522-22 (PMC9241797; doi:10.1128/spectrum.00522-22)
Supplement: SUPPLEMENTAL FILE 1 — Figure S1, S2 and S3. Table S1. Download spectrum.00522-22-s001.pdf, PDF file, 1.1 MB [file spectrum.00522-22-s001.pdf]

Supplementary Document to "The *fruB* Gene of *Streptococcus mutans* Encodes an Endo-levanase that Enhances Growth on Levan and Influences Global Gene Expression"

Brinta Chakraborty, Lin Zeng and Robert A. Burne

Department of Oral Biology, University of Florida College of Dentistry,

Gainesville, Florida, USA

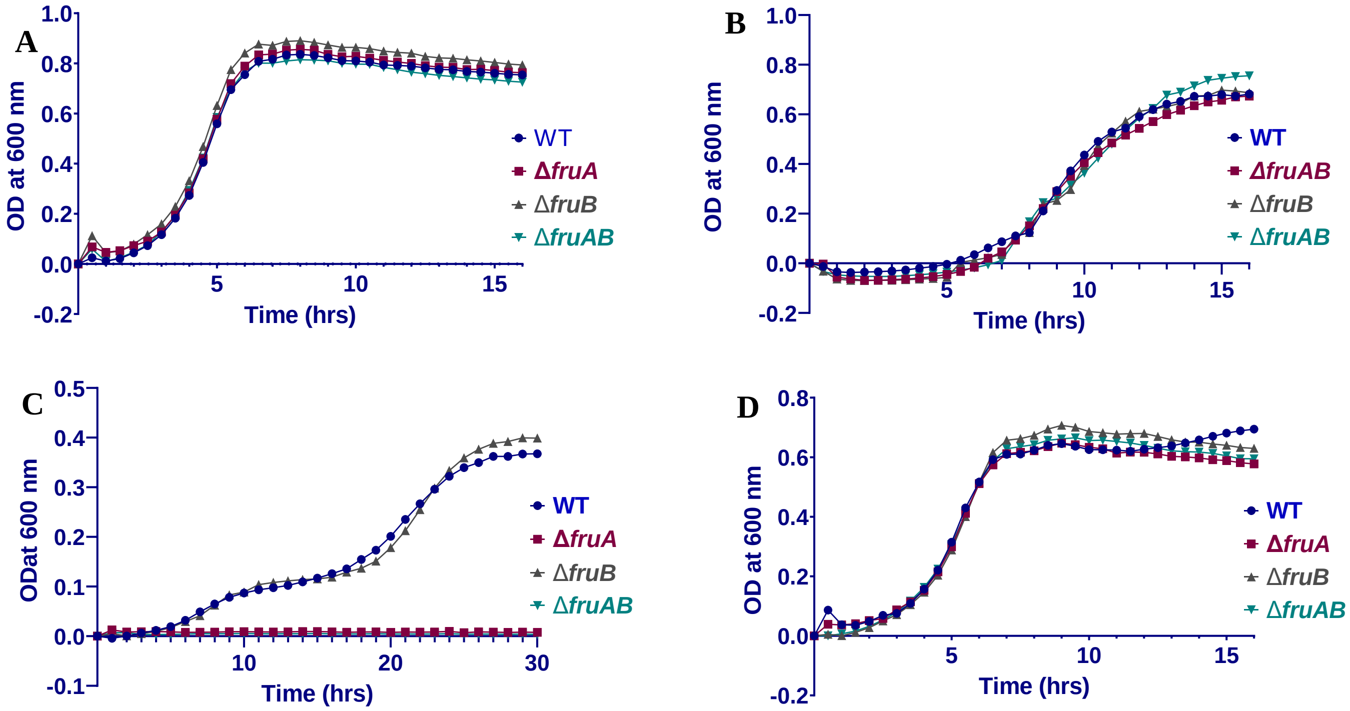

Figure S1. Growth curves of *S. mutans* wild type and *ΔfruA*, *ΔfruB* and *ΔfruAB* strains on different carbohydrate sources. (A) Tryptone vitamin (TV) medium supplemented with 10 mM fructose**, (B)** 10 mM glucose, **(C)** 0.2% inulin and 2 mM fructose, and **(D)** BHI medium.

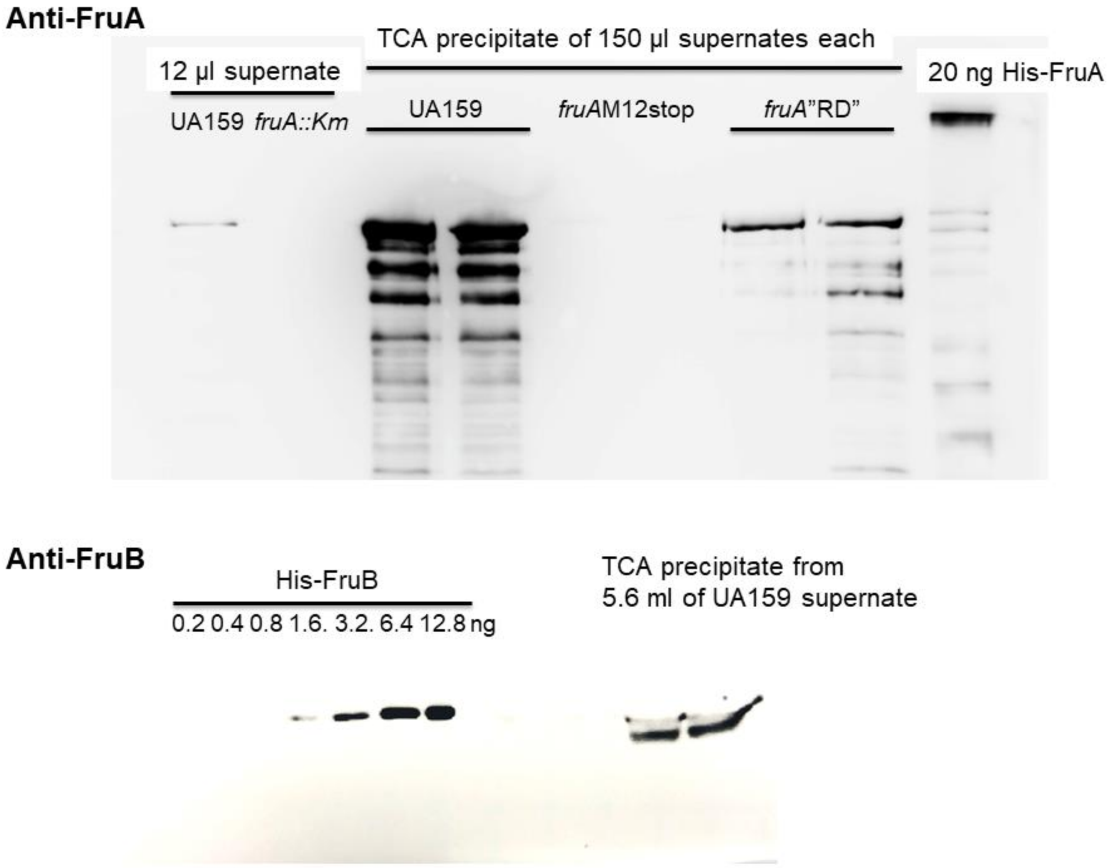

Figure S2. FruA and FruB abundance shown by Western blots. Immunoblotting was carried out using affinity-purified, anti-FruA (top) or anti-FruB (bottom) antiserum against culture supernates of the wild type UA159 and various mutants prepared with TV-inulin. FruA appeared much more abundant than FruB as 12 µl of supernate without TCA treatment (under "Supernate") was sufficient for detection. Estimation based on densitometry of this Western blot put FruA protein copy number at approximately 900/cell, as opposed to 11~26/cell for FruB; a 30~80-fold difference in abundance. *fruA*M12stop is a point mutant of *fruA* that resulted in loss of FruA translation, *fruA*"RD" is a mutant with truncation of the catalytic domain of FruA. Purified recombinant protein His-FruA was loaded as a control and it migrated significantly higher than the wild-type protein in *S. mutans*. The low abundance of the FruA"RD" truncation mutant is caused by insufficient induction of the *fruA* promoter as 0.5% of fructose instead of inulin was used due to loss of growth on inulin, which likely triggered CCR.

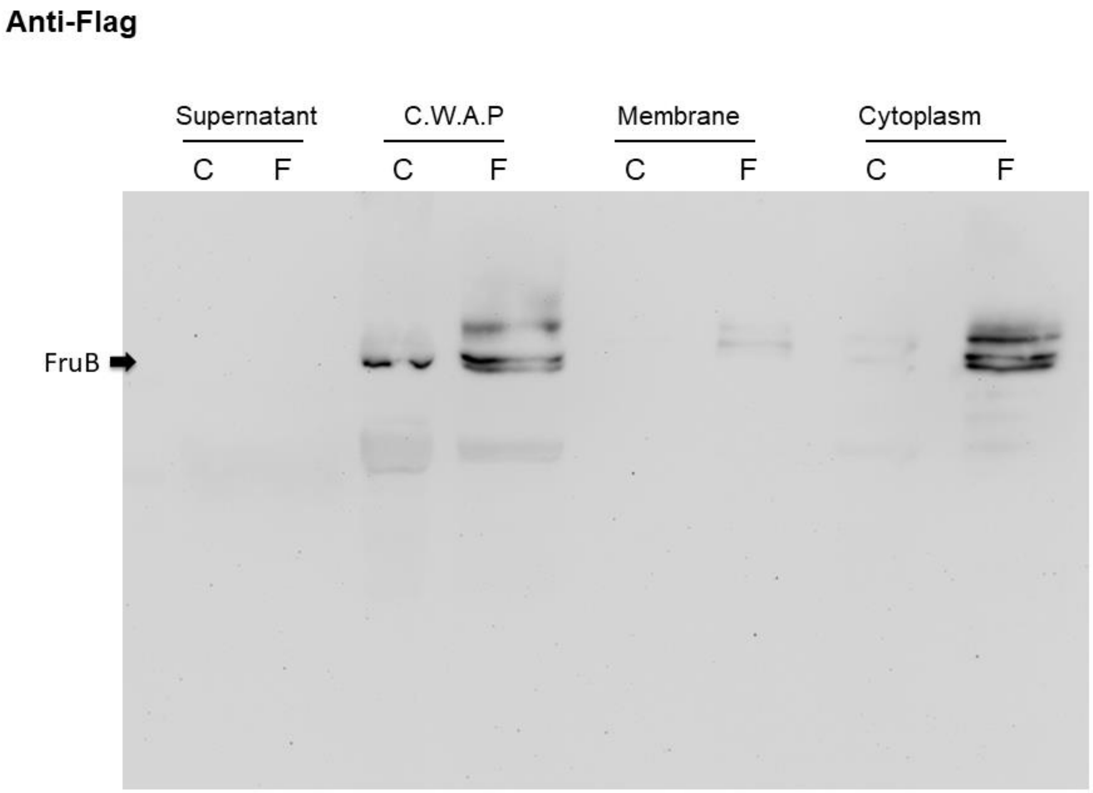

Figure S3. Localization of Flag-tagged recombinant FruB. Western blot with anti-Flag monoclonal antibody (rabbit anti-mouse as secondary). C: UA159; F: FruB-Flag fusion strain; both were cultured in inulin. Signal was detected in cytoplasm and cell wall associated proteins (C.W.A.P).

Table S1. Genes differentially expressed in *fruB* mutant compared to WT UA159

Gene ID	Gene Function	Log2 ∆*fruB*/WT	FDR
**SMU_07**	peptidyl-tRNA hydrolase	1.92	1.43E-18
**SMU_08**	transcription-repair coupling factor	1.83	1.19E-15
**SMU_09**	small RNA binding protein	2.78	1.22E-14
**SMU_10**	cell-division protein DivIC	2.38	2.38E-07
**SMU_11**	Streptococcus-specific protein	1.69	0.00016401
**SMU_12**	conserved hypothetical protein	1.94	8.69E-14
**SMU_13**	cell-cycle protein, PP-loop superfamily	1.53	1.42E-10
**SMU_39**	conserved hypothetical protein	-1.07	0.00211954
**SMU_43**	conserved hypothetical protein (possible site-specific DNA-methyltransferase/restriction modification enzyme)	2.01	7.25E-08
**SMU_44**	conserved hypothetical protein	2.62	7.23E-13
**SMU_45**	hypothetical protein	2.39	5.63E-15
**SMU_46**	hypothetical protein	2.15	0.00011987
**SMU_47**	hypothetical protein	2.05	9.72E-10
**SMU_52**	conserved hypothetical protein	1.27	1.20E-07
**SMU_53**	conserved hypothetical protein	1.15	8.6334E-05
**SMU_54**	amino acid racemase	1.29	1.1749E-05
**SMU_55**	hypothetical protein	1.73	1.76E-08
**SMU_56**	Streptococcus-specific protein	1.95	2.1253E-06
**SMU_58**	hypothetical protein	1.45	1.44E-08
**SMU_63c**	conserved hypothetical protein	1.75	1.30E-22
**SMU_79**	fructan hydrolase; exo-beta-D-fructosidase	-4.84	1.83E-69
**SMU_96**	DNA-directed RNA polymerase, delta subunit	1.44	4.34E-12
**SMU_99**	fructose-bisphosphate aldolase	-1.02	0.0012442
**SMU_102**	PTS system, IID component	1.23	6.1078E-05
**SMU_103**	PTS system, IIA component	1.61	4.11E-07
**SMU_104**	glycosyl hydrolase, alpha-glucosidase	1.67	4.33E-11
**SMU_120**	50S ribosomal protein L28	-1.31	2.04E-09
**SMU_125**	conserved hypothetical protein	-1.04	0.00015526
**SMU_133c**	carbohydrate permease; transmembrane efflux protein	-1.12	4.7503E-05
**SMU_139**	oxalate decarboxylase	-1.01	0.00127343
**SMU_140**	glutathione reductase	-1.09	0.00040832
**SMU_141**	conserved hypothetical protein	-1.21	3.01E-05
**SMU_152**	hypothetical protein	1.59	3.6158E-06
**SMU_153**	hypothetical protein	2.10	3.57E-09
**SMU_162c**	conserved hypothetical protein	1.36	2.51E-10
**SMU_163c**	hypothetical protein	1.37	4.16E-10

Gene ID	Gene Function	Log2 ∆*fruB*/WT	FDR
**SMU_184**	ABC transporter element, iron predicted binding protein	2.11	0.00313516
**SMU_185**	hypothetical protein	2.12	0.00295715
**SMU_191c**	phage-related integrase	1.11	0.00115441
**SMU_198c**	conjugative transposon protein	1.14	6.3065E-05
**SMU_199c**	hypothetical protein	2.01	2.51E-10
**SMU_200c**	hypothetical protein	1.41	0.00041505
**SMU_201c**	conserved hypothetical protein	1.26	5.5067E-05
**SMU_202c**	conserved hypothetical protein/Streptococcus-specific protein	1.39	4.4461E-05
**SMU_204c**	hypothetical protein	1.78	2.90E-07
**SMU_205c**	conserved hypothetical protein	1.13	0.00036885
**SMU_206c**	hypothetical protein	1.37	0.00013065
**SMU_207c**	transcriptional regulator	1.21	9.0092E-06
**SMU_209c**	hypothetical protein	1.31	0.00073285
**SMU_210c**	hypothetical protein	1.12	0.00133386
**SMU_219**	hypothetical protein	1.38	2.80E-08
**SMU_220c**	hypothetical protein	1.26	1.8535E-06
**SMU_221c**	phage-related integrase, truncated	1.84	2.44E-16
**SMU_223c**	hypothetical protein	1.08	0.00021367
**SMU_236c**	transcriptional regulator	-1.31	6.53E-07
**SMU_237c**	ABC transporter multidrug permease protein	-1.88	1.99E-07
**SMU_238c**	ABC transporter, ATP-binding protein	-1.91	4.67E-07
**SMU_287**	ABC transport protein ComB, accessory factor for ComA	1.44	1.37E-14
**SMU_299c**	bacteriocin peptide precursor	-1.81	2.88E-09
**SMU_338**	conserved hypothetical protein, Jag protein	1.04	5.2064E-05
**SMU_353**	thiamine pyrophosphokinase	1.01	0.0001306
**SMU_360**	glyceraldehyde-3-phosphate dehydrogenase; plasmin receptor	-1.08	0.00138028
**SMU_372**	hypothetical protein	1.54	4.75E-08
**SMU_373**	hypothetical protein	1.27	1.161E-06
**SMU_374**	oxidoreductase/ dehydrogenase	1.41	4.10E-09
**SMU_375**	hypothetical protein	1.28	1.1396E-05
**SMU_376**	aminotransferase	1.56	6.46E-13
**SMU_378**	hypothetical protein	1.60	7.47E-08
**SMU_379**	hypothetical protein	1.16	4.9022E-06
**SMU_390**	hypothetical protein	-1.06	0.00499521
**SMU_391c**	conserved hypothetical protein	-1.15	3.432E-05
**SMU_396**	glycerol uptake facilitator protein	-1.08	3.0428E-05
**SMU_419**	conserved hypothetical protein	1.40	2.45E-07
**SMU_420**	ribosomal protein L7A family	1.55	1.072E-05

Gene ID	Gene Function	Log2 △*fruB*/WT	FDR
**SMU_421**	translation initiation factor IF-2	1.17	6.53E-10
**SMU_422**	ribosome binding factor A	1.64	1.91E-13
**SMU_442**	conserved hypothetical protein	-1.08	0.0002314
**SMU_447**	conserved hypothetical protein	1.78	4.13E-14
**SMU_473**	conserved hypothetical protein	1.31	1.32E-12
**SMU_474**	S-ribosylhomocysteine lyase (S-ribosylhomocysteinase)	-1.01	0.000163
**SMU_496**	cysteine synthetase A	-1.21	0.00024987
**SMU_502**	conserved hypothetical protein	1.30	1.19E-09
**SMU_504**	site-specific DNA-adenine methyltransferase	2.13	1.02E-21
**SMU_505**	adenine-specific DNA methylase (DpnIIB)	2.57	8.08E-27
**SMU_506**	type II restriction endonuclease	2.17	2.40E-20
**SMU_526c**	transcriptional regulator	1.01	4.9721E-06
**SMU_532**	anthranilate synthase, component I	1.34	5.65E-07
**SMU_540**	peroxide resistance protein / iron binding protein	-1.08	8.3407E-05
**SMU_549**	undecaprenyl-PP-MurNAc-pentapeptide-UDPGlcNAc GlcNAc transferase	1.18	3.46E-10
**SMU_550**	cell division protein	1.53	1.22E-14
**SMU_554**	conserved hypothetical protein	1.13	7.11E-11
**SMU_555**	conserved hypothetical protein	1.31	3.16E-10
**SMU_556**	conserved hypothetical protein	1.82	3.36E-21
**SMU_557**	cell division initiation protein	1.45	2.95E-17
**SMU_564**	conserved hypothetical protein	-1.15	3.3906E-06
**SMU_574c**	effector of murein hydrolase	-1.10	0.00654374
**SMU_583**	probable hemolysin A	1.31	1.06E-07
**SMU_584**	arginine repressor	1.48	1.13E-08
**SMU_587**	lipase/acylhydrolase with GDSL-like domain	1.25	1.55E-07
**SMU_588**	conserved hypothetical protein	1.32	3.21E-09
**SMU_589**	histone-like DNA-binding protein	-1.18	8.131E-05
**SMU_595**	dihydroorotate dehydrogenase (dihydroorotate oxidase)	-1.16	0.00017926
**SMU_600c**	conserved hypothetical protein	-1.73	1.009E-05
**SMU_602**	sodium-dependent transporter	-2.20	4.11E-07
**SMU_604**	conserved hypothetical protein	1.20	0.0019887
**SMU_605**	hypothetical protein	2.84	6.06E-19
**SMU_606**	hypothetical protein	2.32	6.76E-21
**SMU_616**	hypothetical protein	-1.41	7E-05
**SMU_618**	hypothetical protein	-1.45	1.3896E-05
**SMU_629**	superoxide dismutase	-1.41	1.1813E-06
**SMU_633**	thioesterase	-1.05	3.9336E-06
**SMU_642**	hypothetical protein	1.37	0.00017309
**SMU_643**	acetyl esterase/ sugar hydrolase	1.18	2.3089E-06

Gene ID	Gene Function	Log2 △*fruB*/WT	FDR
**SMU_682**	hypothetical protein	2.52	9.63E-26
**SMU_683**	conserved hypothetical protein	2.07	2.42E-26
**SMU_684**	hypothetical protein	2.62	1.73E-30
**SMU_685**	hypothetical protein	2.83	4.56E-26
**SMU_688**	conserved hypothetical protein	1.82	9.47E-14
**SMU_694c**	ferredoxin	-1.50	1.0202E-05
**SMU_706c**	conserved hypothetical protein	-1.13	1.4053E-05
**SMU_727**	mercury resistance operon negative regulator	1.03	3.1457E-05
**SMU_730**	conserved hypothetical protein	1.80	4.0405E-05
**SMU_731**	ABC transporter, ATP-binding protein	1.72	7.37E-13
**SMU_742**	conserved hypothetical protein, Cof family	1.10	0.00030165
**SMU_743**	hydrolase, haloacid dehalogenase-like family	1.07	0.00031278
**SMU_758c**	conserved hypothetical protein	-1.22	9.46E-09
**SMU_766**	transposase	2.09	0.00210798
**SMU_767**	transposase	1.39	0.00059646
**SMU_768c**	conserved hypothetical protein	1.01	0.00383396
**SMU_790**	conserved hypothetical protein	1.91	0.00011667
**SMU_804**	hypothetical protein	1.72	5.80E-12
**SMU_812**	hypothetical protein	1.00	0.00261132
**SMU_813**	hypothetical protein (possible transcriptional regulator)	1.52	4.79E-12
**SMU_815**	amino acid ABC transporter, periplasmic amino acid-binding protein	1.04	5.1004E-05
**SMU_817**	amino acid ABC transporter, substrate-binding protein	1.06	2.2076E-05
**SMU_838**	glutathione reductase	-1.11	1.5968E-05
**SMU_856**	bifunctional protein: pyrimidine operon regulatory protein and uracil phosphoribosyltransferase	-1.01	0.00014361
**SMU_865**	30S ribosomal protein S16	-1.23	1.6522E-05
**SMU_866**	conserved hypothetical protein	-1.26	1.1472E-06
**SMU_891**	type I restriction-modification system DNA methylase	1.20	5.91E-07
**SMU_892**	type I restriction-modification system specificity determinant	2.26	3.08E-21
**SMU_893**	anticodon nuclease	2.39	3.62E-23
**SMU_895**	conserved hypothetical protein	1.69	2.2471E-05
**SMU_896**	conserved hypothetical protein	1.83	2.54E-08
**SMU_897**	type I restriction-modification system,helicase subunits	2.50	1.51E-30
**SMU_911c**	hypothetical protein	-1.23	4.5245E-05
**SMU_913**	NADP-specific glutamate dehydrogenase	-1.10	2.2076E-05
**SMU_924**	thiol peroxidase	-1.71	1.44E-08
**SMU_929c**	conserved hypothetical protein	-1.54	2.18E-09
**SMU_954**	pyridoxal kinase	-1.17	4.8042E-06

Gene ID	Gene Function	Log2 △*fruB*/WT	FDR
**SMU_955**	conserved hypothetical protein	-1.17	3.06E-07
**SMU_986c**	conserved hypothetical protein	1.01	0.00442369
**SMU_991**	conserved hypothetical protein (similar to ribonucleotide reductase alpha subunit)	-1.12	3.368E-05
**SMU_1011**	citG protein	1.17	8.212E-06
**SMU_1012c**	transcriptional regulator	1.08	0.00010368
**SMU_1013c**	Mg2+/citrate complex transporter	1.12	1.5512E-05
**SMU_1016**	biotin carboxyl carrier protein	1.07	3.368E-05
**SMU_1025**	transcriptional regulator	1.03	0.00441991
**SMU_1026**	conserved hypothetical protein	1.32	0.00040701
**SMU_1027**	transcriptional regulator (TetR/AcrR family)	2.02	4.86E-10
**SMU_1028**	hydrolase or acyltransferase	1.68	1.33E-10
**SMU_1037c**	histidine kinase	1.14	2.27E-07
**SMU_1038c**	response regulator	1.17	1.26E-07
**SMU_1045c**	NAD(+) kinase (ATP-NAD kinase)	1.27	1.93E-08
**SMU_1046c**	GTP pyrophosphokinase	1.79	3.33E-12
**SMU_1063**	amino acid ABC transporter, ATP-binding protein	1.04	6.9147E-06
**SMU_1069c**	hypothetical protein	1.19	4.576E-05
**SMU_1070c**	conserved hypothetical protein	1.08	0.0012031
**SMU_1080c**	conserved hypothetical protein	1.38	2.85E-09
**SMU_1081c**	conserved hypothetical protein	1.15	3.7164E-06
**SMU_1107c**	conserved hypothetical protein	1.27	2.67E-09
**SMU_1116c**	hypothetical protein	1.60	2.16E-11
**SMU_1117**	H2O-forming NADH Oxidase	1.09	1.0648E-05
**SMU_1125c**	conserved hypothetical protein	1.04	0.00142196
**SMU_1126**	pantothenate kinase	1.15	5.8729E-05
**SMU_1127**	30S ribosomal protein S20	-1.07	8.5666E-06
**SMU_1129**	two-component response regulator	1.11	2.5958E-05
**SMU_112c**	transcriptional regulator, RpiR family	1.09	1.3456E-05
**SMU_1133**	phosphate uptake regulatory protein	1.66	2.16E-15
**SMU_1134c**	phosphate ABC transporter, ATP-binding protein	1.36	4.33E-11
**SMU_1154c**	conserved hypothetical protein	1.35	3.42E-11
**SMU_1156c**	hypothetical protein	1.70	9.64E-13
**SMU_1157c**	conserved hypothetical protein	2.13	1.57E-19
**SMU_1158c**	conserved hypothetical protein	1.86	5.25E-20
**SMU_1159c**	hypothetical protein	2.03	9.49E-12
**SMU_1160c**	hypothetical protein	2.30	1.41E-25
**SMU_1161c**	conserved hypothetical protein	1.83	3.10E-15
**SMU_1175**	sodium:alanine (or glycine) symporter	-1.95	4.18E-07
**SMU_1193**	transcriptional regulator, GntR family	1.44	5.56E-08

Gene ID	Gene Function	Log2 △*fruB*/WT	FDR
**SMU_1194**	ABC transporter, ATP-binding protein	1.23	3.64E-09
**SMU_1205c**	hypothetical protein	1.49	4.92E-11
**SMU_1206c**	conserved hypothetical protein	1.62	2.51E-11
**SMU_1207**	cell filamentation / mobilization protein	1.25	5.00E-10
**SMU_1208c**	hypothetical protein	1.48	2.95E-13
**SMU_1209c**	hypothetical protein	1.48	6.23E-12
**SMU_1211**	conserved hypothetical protein	-1.06	0.00025629
**SMU_1249c**	hypothetical protein	1.48	4.33E-11
**SMU_1250c**	hypothetical protein	2.00	1.15E-11
**SMU_1254**	phosphatase	-1.08	8.6312E-05
**SMU_1296**	glutathione S-transferase	-1.33	1.761E-06
**SMU_1298**	50S ribosomal protein L31	-1.41	4.8042E-06
**SMU_1322**	acetoin reductase	-1.06	1.5699E-05
**SMU_1323**	conserved hypothetical protein (possible hydrolase)	-1.08	0.00032294
**SMU_1330c**	transposase, IS1167, fragment	-1.03	0.0046045
**SMU_1334**	phosphopantetheinyl transferase	1.33	3.5267E-05
**SMU_1335c**	enoyl-acyl carrier protein(ACP) reductase; dioxygenase related to 2-nitropropane dioxygenase	1.77	1.95E-12
**SMU_1337c**	alpha/beta superfamily hydrolases	1.32	0.00041505
**SMU_1339**	bacitracin synthetase; surfactin synthetase	3.20	3.87E-46
**SMU_1340**	bacitracin synthetase 1/ tyrocidin synthetase III	3.71	1.51E-52
**SMU_1341c**	gramicidin S synthase/mycosubtilin synthetase chain mycB	3.50	1.05E-45
**SMU_1342**	bacitracin synthetase	3.48	1.89E-47
**SMU_1343c**	polyketide synthase	3.53	1.83E-69
**SMU_1344c**	malonyl CoA-acyl carrier protein transacylase	2.93	2.81E-43
**SMU_1345c**	peptide synthetase similar to mycA	3.42	4.19E-62
**SMU_1346**	thioesterase II-like protein	2.75	4.69E-36
**SMU_1347c**	Permease-ABC-type antimicrobial peptide transport system, Permease component	2.38	2.90E-30
**SMU_1348c**	ABC transporter ATP-binding Protein	2.02	1.69E-13
**SMU_1351**	transposase fragment	1.59	2.87E-11
**SMU_1354c**	transposase	1.19	7.9709E-05
**SMU_1355c**	transposase	1.19	0.00122165
**SMU_1356c**	transposase	1.55	1.6997E-06
**SMU_1360c**	hypothetical protein	1.97	1.01E-12
**SMU_1361c**	transcriptional regulator	2.19	2.87E-19
**SMU_1363c**	transposase fragment (IS605/IS200-like)	1.23	5.12E-07
**SMU_1365c**	Permease-FtsX-like permease	2.34	4.27E-28
**SMU_1366c**	ABC transporter ATP-binding Protein	1.94	1.14E-13

Gene ID	Gene Function	Log2 △*fruB*/WT	FDR
**SMU_1379**	hypothetical protein-HTH XRE domain	3.79	0.00021888
**SMU_1392c**	conserved hypothetical protein; possible acetyltransferase	1.12	1.3006E-05
**SMU_1397c**	possible transcriptional repressor	-1.35	4.1095E-06
**SMU_1399**	hypothetical protein	-2.14	0.00910997
**SMU_1402c**	conserved hypothetical protein	1.04	9.4741E-06
**SMU_1403c**	conserved hypothetical protein	1.05	1.5631E-06
**SMU_1404c**	conserved hypothetical protein	1.20	2.10E-07
**SMU_1405c**	conserved hypothetical protein	2.06	7.70E-23
**SMU_1442c**	conserved hypothetical protein	1.43	1.19E-10
**SMU_1443c**	tributyrin esterase	1.03	9.51E-08
**SMU_1479**	conserved hypothetical protein	-1.24	2.8019E-06
**SMU_1485c**	conserved hypothetical protein (possible endonuclease)	1.07	4.46E-07
**SMU_1502c**	conserved hypothetical protein	-1.31	1.21E-07
**SMU_1508c**	coenzyme PQQ synthesis protein /possible oxidoreductase	1.04	3.8839E-06
**SMU_1509**	transcriptional regulator	1.26	9.11E-08
**SMU_1513**	chromosome segregation SMC protein	1.51	6.75E-17
**SMU_1560**	conserved hypothetical protein	-1.09	0.00010095
**SMU_1574c**	conserved hypothetical protein	1.82	3.67E-11
**SMU_1575c**	hypothetical protein	1.62	0.00035979
**SMU_1576c**	hypothetical protein	1.92	1.1813E-06
**SMU_1577c**	conserved hypothetical protein	1.51	6.84E-09
**SMU_1602**	NAD(P)H-flavin oxidoreductase	-1.33	1.1491E-05
**SMU_1603**	lactoylglutathione lyase	-1.51	9.42E-07
**SMU_1604c**	conserved hypothetical protein	1.14	0.00049711
**SMU_1609c**	preprotein translocase, SecG subunit	-1.01	3.6357E-06
**SMU_1628**	conserved hypothetical protein	-1.32	1.3456E-05
**SMU_1642c**	conserved hypothetical protein	1.15	2.97E-07
**SMU_1643c**	conserved hypothetical protein	1.17	0.00096135
**SMU_1644c**	conserved hypothetical protein	1.12	0.00330123
**SMU_1648c**	hypothetical protein	-1.12	1.8675E-05
**SMU_1649**	exodeoxyribonuclease III/ Smx nuclease	-1.11	0.00037979
**SMU_1650**	endonuclease III (DNA repair)	-1.16	0.0001169
**SMU_1657c**	nitrogen regulatory protein PII	-1.54	3.0894E-06
**SMU_1700c**	LrgB-like protein; possible murein hydrolase regulator	-1.68	4.38E-07
**SMU_1701c**	conserved hypothetical protein	-1.76	1.66E-07
**SMU_1711**	ribosomal large subunit pseudouridine synthase B	1.13	2.0246E-06
**SMU_1712c**	segregation and condensation protein B	1.20	2.7075E-05
**SMU_1713c**	segregation and condensation protein A	1.05	0.00013065

Gene ID	Gene Function	Log2 △*fruB*/WT	FDR
**SMU_1728**	transcription elongation factor GreA	1.89	1.56E-17
**SMU_1729c**	aminodeoxychorismate lyase-like protein (fragment)	1.85	7.70E-23
**SMU_1730c**	arylalkylamine n-acetyltransferase	1.36	4.66E-11
**SMU_1752c**	hypothetical protein	-1.13	1.7884E-05
**SMU_1758c**	conserved hypothetical protein	1.13	1.43E-07
**SMU_1760c**	conserved hypothetical protein	1.89	1.30E-16
**SMU_1761c**	conserved hypothetical protein	2.35	7.78E-22
**SMU_1762c**	conserved hypothetical protein	2.32	3.78E-20
**SMU_1763c**	conserved hypothetical protein	1.88	4.89E-14
**SMU_1764c**	conserved hypothetical protein	1.81	2.75E-14
**SMU_1771c**	hypothetical protein	1.15	0.0026122
**SMU_1772c**	hypothetical protein	2.05	1.68E-11
**SMU_1773c**	conserved hypothetical protein	1.54	4.10E-09
**SMU_1775c**	hypothetical protein	1.66	1.50E-08
**SMU_1780**	RecA regulator RecX	1.67	1.10E-11
**SMU_1827**	biotin biosynthesis protein	-1.22	0.00555715
**SMU_1828**	universal stress protein family	-1.07	1.2645E-05
**SMU_1869**	thioredoxin	-1.06	4.01E-08
**SMU_1889c**	hypothetical protein (possible relation to bacteriocin BlpU)	-1.26	0.00011409
**SMU_1897**	ABC transporter, ATP-binding protein; similar to BlpA	-1.01	0.00035319
**SMU_1900**	ABC transporter	1.16	0.00694273
**SMU_1903c**	hypothetical protein	2.70	8.78E-19
**SMU_1904c**	hypothetical protein	2.77	1.00E-29
**SMU_1905c**	hypothetical protein	2.78	4.15E-28
**SMU_1906c**	bacteriocin-related protein	2.01	7.75E-20
**SMU_1908c**	hypothetical protein	2.23	6.06E-19
**SMU_1909c**	hypothetical protein	2.73	6.89E-28
**SMU_1910c**	hypothetical protein	3.19	1.33E-45
**SMU_1912c**	hypothetical protein	3.07	1.83E-50
**SMU_1913c**	hypothetical protein; immunity protein, BLpL-like	2.55	1.31E-24
**SMU_1922**	chromosome replication initiation and membrane attachment protein	1.44	2.80E-08
**SMU_1923c**	conserved hypothetical protein	1.07	1.4324E-05
**SMU_1927**	PsaA protein/ ABC transporter, ATP-binding protein	1.74	5.66E-13
**SMU_1928**	protein secretion ABC transport permease	1.79	1.00E-15
**SMU_1941**	ABC transporter solute-binding protein	1.26	3.46E-09
**SMU_1969c**	probable transcriptional regulator	1.29	1.42E-07
**SMU_2002**	30S ribosomal protein S11	1.08	4.8663E-05
**SMU_2003**	30S ribosomal protein S13, N-terminal fragment	1.06	0.00019621

Gene ID	Gene Function	Log2 △*fruB*/WT	FDR
**SMU_2059c**	conserved hypothetical protein; possible membrane protein	-1.38	0.00010017
**SMU_2061**	hypothetical protein	1.21	6.18E-07
**SMU_2085**	recombinase A	1.26	2.09E-13
**SMU_2086**	competence damage-inducible protein A	1.44	1.55E-15
**SMU_2087**	3-methyladenine DNA glycosylase I	1.34	4.87E-08
**SMU_2088**	Holliday junction DNA helicase	1.18	3.65E-07
**SMU_2096c**	conserved hypothetical protein	1.21	0.00124779
**SMU_2113c**	conserved hypothetical protein	1.62	6.27E-11
**SMU_2114c**	probable transcriptional regulator, MerR family	1.08	0.00017515
**SMU_2135c**	30S Ribosomal protein S4	-1.09	0.00070484
**SMU_2137c**	conserved hypothetical protein	1.42	8.89E-08
**SMU_2138**	replicative DNA helicase	1.16	2.86E-09
**SMU_2139c**	50S ribosomal protein L9	2.10	2.40E-20
**SMU_2147c**	conserved hypothetical protein	1.39	4.98E-13
**SMU_2150c**	ABC transporter, ATP-binding protein	1.09	6.4044E-06
**SMU_2152c**	conserved hypothetical protein	1.33	2.87E-11
**SMU_2153c**	peptidase, M16 family	1.91	6.02E-18
**SMU_2154c**	peptidase, M16 family	1.03	3.3352E-06
**SMU_2155**	conserved hypothetical protein	1.02	3.0316E-06
**SMU_2156**	recombination protein F (DNA replication and repair ATPase)	1.16	6.31E-08
**SMU_2164**	serine protease	1.62	9.64E-15
**SMU_2165**	chromosome segregation protein, ParB-like nuclease domain	1.74	4.13E-14
